# Meta-analysis of primary target genes of peroxisome proliferator-activated receptors

**DOI:** 10.1186/gb-2007-8-7-r147

**Published:** 2007-07-25

**Authors:** Merja Heinäniemi, J Oskari Uski, Tatjana Degenhardt, Carsten Carlberg

**Affiliations:** 1 Department of Biochemistry, University of Kuopio, FIN-70211 Kuopio, Finland; 2 Life Sciences Research Unit, University of Luxembourg, L-1511 Luxembourg

## Abstract

**Background:**

Peroxisome proliferator-activated receptors (PPARs) are known for their critical role in the development of diseases, such as obesity, cardiovascular disease, type 2 diabetes and cancer. Here, an *in silico *screening method is presented, which incorporates experiment- and informatics-derived evidence, such as DNA-binding data of PPAR subtypes to a panel of PPAR response elements (PPREs), PPRE location relative to the transcription start site (TSS) and PPRE conservation across multiple species, for more reliable prediction of PPREs.

**Results:**

*In vitro *binding and *in vivo *functionality evidence agrees with *in silico *predictions, validating the approach. The experimental analysis of 30 putative PPREs in eight validated PPAR target genes indicates that each gene contains at least one functional, strong PPRE that occurs without positional bias relative to the TSS. An extended analysis of the cross-species conservation of PPREs reveals limited conservation of PPRE patterns, although PPAR target genes typically contain strong or multiple medium strength PPREs. Human chromosome 19 was screened using this method, with validation of six novel PPAR target genes.

**Conclusion:**

An *in silico *screening approach is presented, which allows increased sensitivity of PPAR binding site and target gene detection.

## Abstract 2

A combined experimental and *in silico *approach identifies Peroxisome Proliferator Activated Receptor (PPAR) binding sites and six novel target genes in the human genome.

## Background

Lipid level dys-regulation is a characteristic common to some of the most prevalent medical disorders, including obesity, cardiovascular disease and type 2 diabetes [1]. Nuclear receptors (NRs) are transcription factors that have important roles in these diseases, because many of them have lipophilic compounds as ligands, including cholesterol, fatty acids and their metabolic derivatives [2]. For example, native and oxidized polyunsaturated fatty acids as well as arachidonic acid derivatives, such as prostaglandins and prostacyclins, selectively bind the NRs peroxisome proliferator-activated receptor (PPAR)α, PPARγ and PPARβ/δ and stimulate their ability to activate target genes transcriptionally [3]. The PPAR transcription factors are prominent players in the metabolic syndrome, because of their role as important regulators of lipid storage and catabolism [4]. However, they also regulate cellular growth and differentiation and, therefore, have an impact on hyper-proliferative diseases, such as cancer [5]. Known primary PPAR targets may be incompletely characterized for their regulatory regions involved in their regulation by PPARs. In rodents a large number of significantly inducible PPAR target genes have been identified [6,7], while in human cell lines only a few genes are activated more than two-fold by PPAR ligands [8]. In parallel, PPARs have a relatively high basal activity [9]. These facts suggest that there is a need to identify new PPAR response elements (PPREs) and target genes in an unbiased way that is independent of ligand binding and encompasses the whole human genome sequence.

The *in silico *searching of the genome sequence provides another way to identify target genes. An essential prerequisite for the direct modulation of transcription by PPAR ligands is the location of at least one activated PPAR protein close to the transcription start site (TSS) of the respective primary PPAR target gene. This is commonly achieved through the specific binding of PPARs to a PPRE and DNA-looping towards the TSS [10]. In detail, the DNA-binding domain of PPARs contacts the major groove of a double-stranded hexameric DNA sequence with the optimal AGGTCA core binding sequence. PPARs bind to DNA as heterodimers with the NR retinoid X receptor (RXR) [11]. PPREs are therefore formed by two hexameric core binding motifs in a direct repeat orientation with an optimal spacing of one nucleotide (DR1), where PPAR occupies the 5'-motif [12]. However, characterization of PPREs from regulated gene promoters has resulted in a large collection of PPREs that deviate significantly from this consensus sequence. The ubiquity of such PPRE-like sequences on a whole genome level is in contrast to the number of potential PPAR target genes in a physiological context (a few hundred to a few thousand per tissue [13] and the number of receptor molecules (a few thousand per cell). A recent effort to better model the binding preferences of PPARs used position weight matrices to describe all published PPREs [14]. However, such an approach has limited ability to predict *bona fide *PPAR binding *in vivo*.

In addition to binding strength, a number of additional parameters could influence the functionality of a PPRE. One common trend in location of transcription factor binding sites is a positional bias towards the TSS. This would be apparent from the collection of identified PPREs, but is in contrast with a multi-genome comparison of NR binding site distribution [15]. Furthermore, a common approach for the detection of functional binding sites is to rely on conservation. However, maintenance of responsiveness may not require conservation of exact binding site composition. In contrast, there is also evidence to indicate that regulatory regions may evolve with more flexible constraints. Such a stabilizing model of evolution was proposed based on conservation patterns in the *Drosophila eve *gene enhancer, where patterns and locations of binding sites were shown to be divergent, but maintain identical patterns of expression [16]. This turnover has been studied with computer simulations demonstrating that appearance and fixation of novel binding sites occurs in short evolutionary time frames [17].

In this study, we performed an experiment-based informatics approach for the reliable identification of PPREs and PPAR target genes. We chose to take an unbiased approach for the characterization of PPRE binding variants, utilizing an experimental binding strength dataset. As a first step, we performed *in silico *screening and binding strength prediction of PPREs in eight known PPAR target genes and found for each four to nine PPREs within a 10 kB distance of their respective TSSs. Seventeen of these (in total 23) genomic regions were found to be functional in liver- and kidney-derived cells and 12 of them associated with PPARα and its partner proteins. Three of these regions are located in the *uncoupling protein 3 *(*UCP3*) gene, for which so far no PPREs had been identified. Next a collection of 38 validated PPAR target genes in human was used for the detection of features of binding site composition in these genes. In conclusion, significant diversification of binding site composition between species was often observed. However, typically these genes contain strong or multiple medium strength PPREs. Based on this insight, we screened the whole of human chromosome 19 (containing 1,445 annotated genes) and the corresponding syntenic regions in the mouse genome (956 known orthologs) and found that our PPAR responsiveness criteria were passed by 116 genes in both species. Under more stringent criteria 8.7% of human genes in the same chromosome would likely be PPAR targets. All six genes, chosen to be representative from this panel, were shown to be primary PPARα targets. For one of these, the *longevity-assurance homologue 1 *(*LASS1*) gene, we demonstrate that a genomic region containing two PPREs is functional and recruits PPARα as well as its partner proteins.

## Results

### A PPRE binding strength prediction scheme

**Figure 1:**
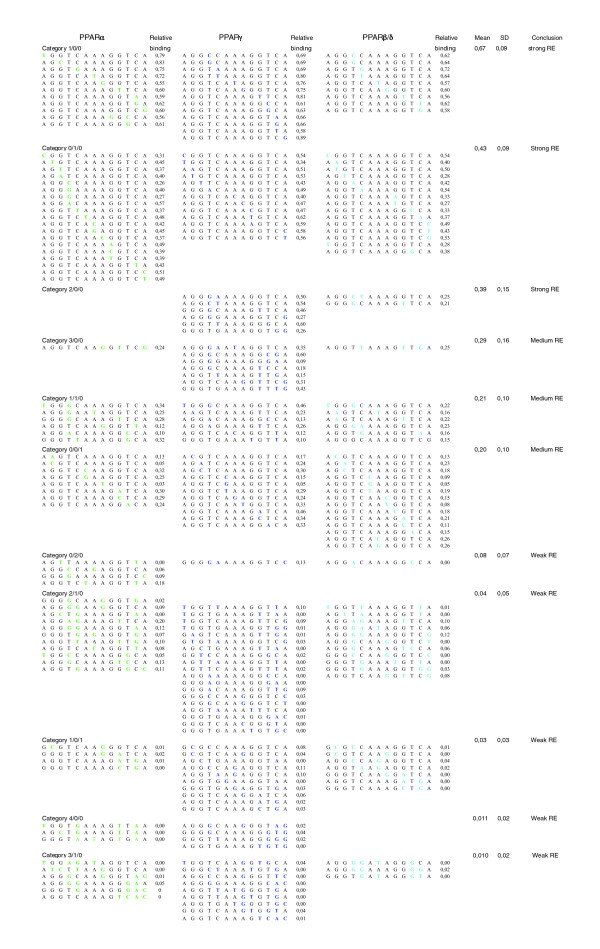
Testing the RE classification scheme on natural DR1-type sequences. The average binding strength of *in vitro *translated PPAR-RXR heterodimers to DR1-type PPREs was determined by gelshift assays in reference to the consensus PPRE AGGTCAAAGGTCA, including all categories (that is, combinations of the classes I, II and III) that resulted in an average binding of at least 1%. Variations from the consensus PPRE are highlighted in green for PPARα, in dark blue for PPARγ and in light blue for PPARβ/δ. In total, the *in vitro *binding data of 136 different REs were used (the non-binding DR1-type REs are shown in Additional data file 1), with a minimum of six sequences for each category. SD, standard deviation.

Recently, we characterized the *in vitro *binding preferences of the three PPAR subtypes on a panel of 39 systematic single nucleotide variations of the consensus DR1-type PPRE (AGGTCAAAGGTCA) [18]. Based on this analysis we subdivided the single nucleotide variants into three classes (Table 1). Sequences in class I are bound by the PPAR subtypes with a strength of 75 ± 15% of that of the consensus PPRE; sequences in class II are bound with a strength of 45 ± 15% of that of the consensus PPRE; and sequences in class III are bound with a strength of 15 ± 15% of that of the consensus PPRE. Although the overall binding pattern of the three PPAR subtypes showed no major differences, some variations gave rise to a PPAR subtype-specific classification. We observed that the number and class of variations seem to correlate with experimental binding. Therefore, we decided to take the concept further to create a classifier for PPREs based on binding data. We sorted a total of 136 DR1-type response elements (REs; including combinations of multiple variations) according to the number and class of variations (Figure 1). The *in vitro *binding strength to these REs in relation to the consensus DR1-type PPRE was determined by gelshift assays for the RXR heterodimers of all three PPAR subtypes. For each category in Figure 1 the average of the relative binding strength was determined (based on 6 to 47 RE/PPAR subtype combinations). REs with 1/0/0, 2/0/0 and 0/1/0 variations (where the numbers indicate the number of variations for the classes I, II and III, respectively) bound the receptor strongly (67%, 43% and 39% relative binding, respectively), REs with 3/0/0, 1/1/0 and 0/0/1 variations were medium PPREs (29%, 22% and 20%, respectively) and REs with 0/2/0, 2/1/0, 1/0/1, 3/1/0 and 4/0/0 variations were considered to be weak PPREs (8%, 4%, 3%, 1% and 1%, respectively). We set 1% as a cut-off limit. Representative DR1-type REs with increasing numbers of more drastic variations were examined as well (Additional data file 1), but these elements were not considered as functional PPREs. Please note that the published PPRE of the *acyl-CoA oxidase 1 *(*ACOX1*) gene [19] belongs to the latter list.

**Table 1:** Systematic variation from consensus DR1-type PPRE

Percent binding strength	PPRE position												
	
	1	2	3	4	5	6	7	8	9	10	11	12	13
**PPARα**													
Consensus (90-100)	A/G	G	G	T	A/C	A	A	A	G	G	T	C	A
Class I (60-90)	T		C		G		T	G		T	C/G	A/G	G
Class II (30-60)	C	T	A/T	A/C/G	T	T	C/G	C	A/C/T			T	C/T
Class III (0-30)		A/C				C/G		T		A/C	A		
													
**PPARγ**													
Consensus (90-100)	A/G	G	G	T	C/G	A	A	A	G	G	T	C	A
Class I (60-90)				C/G	A/T		T	G		T	C/G	A/G/T	G
Class II (30-60)	C/T	A/T	T	A			C	C	A/C/T				C/T
Class III (0-30)		C	A/C			C/G/T	G	T		A/C	A		
													
**PPARβ/δ**													
Consensus (90-100)	A/G	G	G	T	C	A	A	A	G	G	T	C	A
Class I (60-90)				C/G	G/T		T	G		T		G/T	
Class II (30-60)	C	A/T	T	A	A				A/T		C/G	A	G/C/T
Class III (0-30)	T	C	A/C			C/G/T	C/G	C/T	C	A/C	A		

The binding strengths of *in vitro *translated PPAR-RXR heterodimers to 39 systematic variations of the DR1-type consensus PPRE AGGTCAAAGGTCA were determined by gelshift assays in reference to this consensus PPRE. Based on their average binding strength, all variations are sorted into three classes.

The performance of the classifier in predicting novel binding sites was simulated by random sampling of the experimental data in Figure 1 and Additional data file 1 into a training set that was used to re-calculate the category averages at each initialization (approximately 10% of data was used in training) and a validation set that can be used in testing (rest of the data). Representative data from 10 rounds of simulation are shown in Additional data file 2. Interestingly, the category averages were relatively robust to changes in the set of sequences used to calculate the average. This suggests that the introduction of further sequences that belong to the same category will not drastically affect the classifier performance.

### Comparison of PPRE classifier to matrix methods

In order to compare the classifier to the traditional matrix methods, we created a position-specific weight matrix (PSWM) and a position-specific affinity matrix (PSAM) using the PPARγ data from Figure 1 and Additional data file 1. For the PSWM we took all medium and strong PPREs with multiple variations from Figure 1, calculated base pair frequencies and converted these to matrix values by logarithmic transformation, where an equal background frequency was assumed and a pseudocount of 0.01 was included for non-observed base-pairs (bp). We chose not to include the systematic single nucleotide variation screen data, since this would have biased the matrix strongly towards the consensus PPRE. In total, 20 sequences were used to construct the matrix, which is in the order of known binding sites typically used as a basis of such matrices in databases, such as JASPAR or TRANSFAC. The PSAM was chosen to represent a matrix method utilizing the single nucleotide screening data, in order to see if these data are sufficient to capture the binding preferences of multiple variation data.

**Figure 2:**
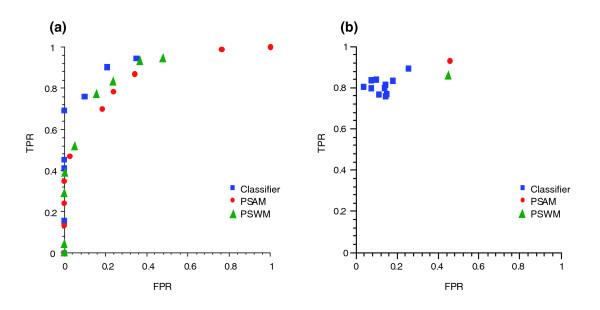
ROC curves comparing *in silico *methods. **(a) **A PSWM constructed from 20 medium and strong PPREs that contain multiple variations, and a PSAM constructed using the single nucleotide data and ten initializations of PPRE classifier created based on Table 1 and random sampling of Figure 1 and Additional data file 1 were compared for their ability to detect binding. True positive rates (TPRs) and false positive rates (FPRs) were calculated, with false positives given when no binding was detected despite prediction, and false negatives given when binding was detected but not predicted (correlation of matrix scores to predicted binding was done based on lines fitted to correlation plots shown in Additional data file 3). A line of no discrimination is a diagonal line and optimum performance approaches the value (0, 1). For clarity, only one representative instance of a PPRE classifier is shown in (a). **(b) **To assess how good the predicted experimental binding estimates were, the performance of the method used was tested with a 15% tolerance interval for a match to experimental binding (5% when prediction was 15% or less) using a single cut-off (the optimal cut-off was 3% for the classifier, 25% or a score of 0.0000015 for PSAM, and 20% or a score of 4.7 for PSWM) and calculating again the FPR and TPR for each method. False positives in this case represented predictions that were too high and false negatives predictions that were too low.

We compared the three methods first on the level of their ability to detect binding. True positive and false positive rates (TPRs and FPRs, respectively) were calculated using different cut-off values for each method and are represented in the form of a receiver operating characteristic (ROC) curve (Figure 2a). The line of no discrimination is indicated as a diagonal line; perfect performance would give a TPR of 1 and FPR of 0. For all methods an optimum performance was detected with FPR from 20-30% and TPR varying from around 90% for the PPRE classifier to 75% for the PSAM. For clarity, one representative classifier curve out of ten calculated is shown.

Next we wanted to know whether the scores correlated with experimental binding when comparing single and multiple variation data. We examined this with correlation plots using the PPARγ data as shown in Additional data file 3. In parallel, we set a tolerance interval of 15% relative to the consensus sequence for a match between predicted binding strength and experimental binding (5%, if the predicted binding was less than 15%) and calculated predictions by the different methods. The equations of the lines fitted to the single nucleotide data (Additional data file 3) were used to correlate matrix scores with binding strength. The ideal cut-off values based on the ROC curves were used in the scoring and produced respective data points in the ROC space (Figure 2b), this time with TPR reflecting correct predictions (no underestimation, if 1) and FPR reflecting overestimated values. Several data points are given for the classifier, representing ten separate initializations with the sampling of training and validation sets.

When comparing the performance of the PSWM between the different datasets (Additional data file 3), a rather clear distinction between the scores of single nucleotide variations (medium and strong PPREs) and the non-binding PPRE classes was observed. The partition of single nucleotide data into two groups of data points shows that the matrix handles variations that were not included in the PPRE set by penalizing these with a constant negative score. Values above 6 still separated quite well from the data points of the last panel. However, the multiple variation data that include weak to strong PPREs were not well resolved by the matrix. Instead a large amount of weak binding sites received high matrix scores, which seems to cause the high FPR rate.

Despite the fit to the single nucleotide data, the PSAM did not offer a significant improvement to the prediction of multiple variations and also had problems differentiating the non-binding PPREs. This is evident by examining the data points between matrix values 0.000001 and 0.000002. This interval includes weak to strong PPREs with identical matrix scores leading to an increased FPR rate. The classifier correlation was weaker for single nucleotide data compared to the PSAM, but the same variation was preserved for multiple variation data. A clear separation between weak PPREs and those of medium and strong strength was achieved. The ability to use a PPRE prediction that also correlates with binding strength is a clear advantage for the evaluation of putative binding site content of target genes. Based on the different comparisons, we chose the PPRE classifier as most suited for the follow-up analysis of PPAR target genes.

### *In silico *analysis of known PPAR target genes

We tested the performance of our PPRE binding strength prediction scheme on eight primary PPAR target genes. We selected the well-known up-regulated human genes *ACOX1 *[19], *carnitine palmitoyl transferase *(*CPT*) *1B *[20] and *PPARα *[21] and the established down-regulated gene *apolipoprotein *(*APO*) *C3 *[22]. The genes *angiopoietin-like 4 *(*ANGPLT4*) [23], *sulfotransferase *(*SULT*) *2A1 *[24] and *Rev-ErbAα *(*RVRα*) [25] were chosen because their PPREs were at unusual positions, such as in an intron or more than 5 kB upstream of their TSS, or of unusual structure, such as a direct repeat with two intervening nucleotides (DR2). Finally, the gene *UCP3 *[26] was included, because despite being an established PPAR target, no PPRE had yet been characterized within its previously studied regulatory regions. Therefore, the latter gene was a specific challenge to our PPRE prediction approach. By real-time quantitative PCR we confirmed the inducibility of all eight genes by PPAR ligands (Additional data file 4) and demonstrated in parallel that our experimental systems, the human cell lines HEK293 (embryonal kidney) and HepG2 (hepatocarcinoma), with the exception of the *APOC3 *gene in HEK293 cells, are well suited for the investigation of these genes.

**Figure 3:**
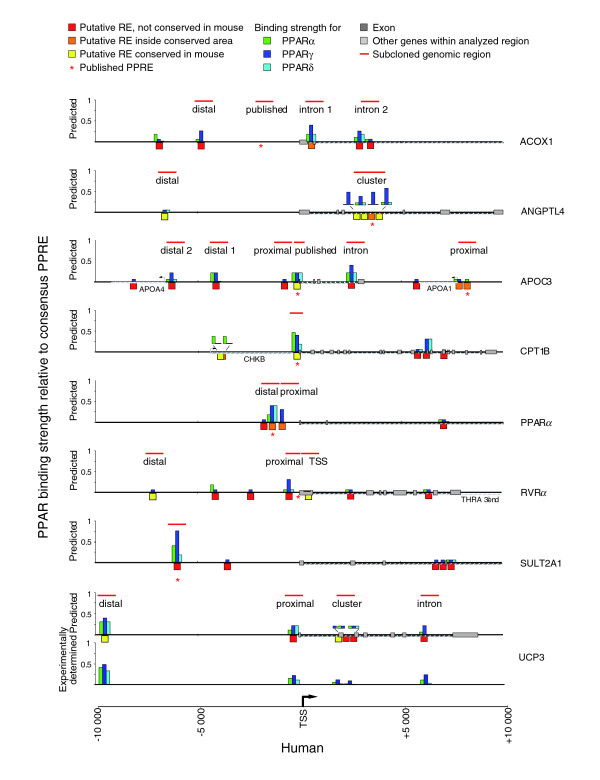
*In silico *analysis of selected primary PPAR target genes. Overview of the genomic organization of eight human PPAR target genes; 10 kB upstream and downstream of the TSSs are shown (horizontal black line). Putative REs (red boxes, no conservation; orange boxes, within conserved area; yellow boxes, conserved) were identified using the classifier by *in silico *screening of the genomic sequences and are classified according to their degree of conservation compared to the orthologous mouse gene. Already published PPREs are indicated by an asterisk. For each predicted RE the calculated binding strengths of PPARα (green), PPARγ (dark blue) and PPARβ/δ (light blue) in reference to a consensus DR1-type PPRE are represented by column height. All putative PPRE sequences are available on request. For the *UCP3 *gene REs, the average *in vitro *DNA binding strength of PPAR-RXR heterodimers was also determined by gelshift experiments and is shown in the same color code scheme. Horizontal red bars indicate the genomic regions that were subcloned for reporter gene assays (Figure 4) and were analyzed by ChIP assays (Figure 5).

For the eight PPAR target genes we performed an *in silico *PPRE search, which spanned 10 kB upstream and downstream of the respective TSS (Figure 3). All PPRE categories that included PPREs with 5% or more binding strength for each subtype are shown. The categories resulting in 1-5% of binding (1/0/1, 3/1/0 and 4/0/0) were indicated only when the PPREs were conserved in the mouse genome. Based on sequence alignments of the human and mouse genome, the evolutionary conservation of all putative REs was evaluated on the level of the RE itself and the level of its flanking sequence (± 50 bp). As a result, we found 5 REs in each of the genes *ACOX1*, *CPT1B*, *SULT2A1 *and *ANGPTL4*, 9 in the *APOC3 *gene, 4 in the *PPARα *gene, 7 in the *RVRα *gene and 6 in the *UCP3 *gene, giving rise to a total of 46 REs in the 160 kB genomic sequence examined. The distribution of the putative REs, relative to the TSS, was roughly equal, since 21 and 25 were found in the upstream regions and downstream areas, respectively. In a cross-species comparison (mouse to human), 10 of the 46 REs were found to be evolutionarily conserved and a further 6 REs were located in conserved regions. Our *in silico *screening found the published PPREs of the genes *ANGPTL4*, *APOC3 *and *CPT1B *as evolutionarily conserved REs and the published PPREs of the genes *SULT2A1 *and *PPARα *as non-conserved. As mentioned above, the published RE of the *ACOX1 *gene did not pass our *in silico *screening parameters and we confirmed by gelshift assays that it does not bind PPARs (Additional data file 1). This observation concurs with a previous report [27]. However, in that study it was claimed that the human *ACOX1 *gene may not be an active PPAR target, whereas here we show that the gene is regulated by PPARs and suggest five new binding sites, of which one is located in an evolutionarily conserved area of intron 1.

The *in silico *binding strength predictions were confirmed by gelshift assays for the six REs of the *UCP3 *gene (novel sequences that had not been used for average calculations in Figure 1). Comparing the experimentally determined and the calculated values, all predicted binding sites match the experimentally determined binding strength with a deviation of less than 15%.

Taken together, *in silico *screening predicts that, for each of the eight tested PPAR target genes, there are four to nine PPREs within 10 kB of their respective TSSs, of which at least one is a strong PPRE. The example of the *UCP3 *gene demonstrates the good correlation between *in silico *prediction of PPREs and actual *in vitro *binding of PPAR-RXR heterodimers.

### Functionality of PPAR responsive genomic regions

**Figure 4:**
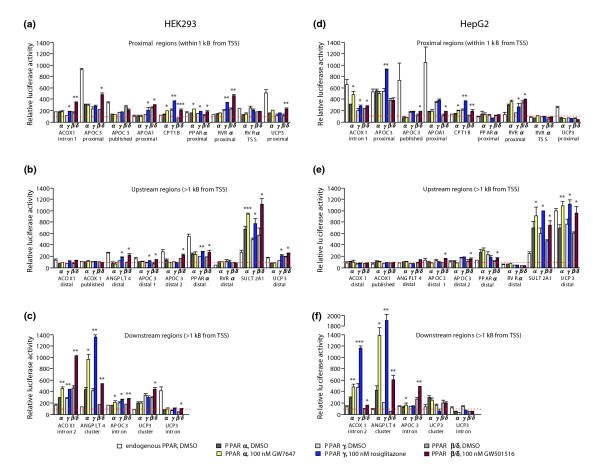
Extra-genomic functionality of the PPRE-containing promoter regions of PPAR target genes. Reporter gene assays were performed with extracts from **(a-c) **HEK293 and **(d-f) **HepG2 cells that were transiently transfected with *luciferase *reporter constructs containing genomic regions of eight human PPAR target genes (please note that the *APOC3 *gene forms a cluster with the genes *APOC1 *and *APOC4*). These were co-transfected with empty expression vector (endogenous PPAR) or the indicated expression vectors for PPARα, PPARγ and PPARβ/δ. Cells were then treated for 16 h with solvent or PPAR subtype-specific ligands. Relative luciferase activity was determined and normalized to the activity of empty cloning vector control co-transfected with empty expression vector (dashed horizontal red line). The genomic regions were subdivided according to their location into close to TSS (a, d), upstream of TSS (b, e) and downstream of TSS (c, f); for further details see Figure 3 and Table 2. Columns represent the means of at least three experiments and bars indicate standard deviations. Two-tailed Student's *t*-tests were performed to determine the significance of the ligand induction in reference to solvent controls (*p < 0.05, **p < 0.01, ***p < 0.001).

We selected within the regulatory regions of the eight PPAR target genes 10 proximal REs (within 1 kB of the TSS), 10 REs further upstream and 10 REs further downstream (the element of the *APOA1 *promoter element was counted as a proximal RE because the gene is a known responding gene [28]). These REs are contained within 23 genomic regions (each approximately 300 bp in length; for locations see Figure 3 and Table 2), which we cloned by PCR and fused with the *thymidine kinase *promoter driving the *luciferase *reporter gene. We included the *ACOX1 *published region, in which we do not predict a PPRE, as a negative control. The activity of the constructs in the absence or presence of PPAR subtype expression vectors in response to PPAR subtype-specific ligands was tested by reporter gene assays in HEK293 and HepG2 cells (Figure 4). Nine of the genomic regions are located within 1 kB of their respective TSSs (Figure 4a,d). With the exception of the *RVRα *gene TSS, which contains a reported DR2-type PPRE, eight of these regions displayed, in at least one of the two cell lines, significant inducibility by PPAR ligands. The region of the human *CPT1B *gene was inducible by all three PPAR subtype-specific ligands in both cell lines, whereas the seven other regions show PPAR subtype- and cell type-specific profiles. An increase in the basal activity compared to empty cloning vector and its subsequent loss due to PPAR over-expression were observed with the proximal regions of the genes *APOC3 *and *UPC3 *in both cell lines as well as in HepG2 cells with the intron 1-containing region of the *ACOX1 *gene and the proximal region of the *APOA1 *gene. This effect may reflect the attraction of constitutively active transcription factors, such as other nuclear receptors that recognize DR1-type REs, for example, HNF4α, to the respective genomic regions and their subsequent displacement [22]. The cellular context may permit stronger activation by the displaced transcription factor, for example, due to higher expression of favored coregulator interaction partners. This switching of activating transcription factor to the binding site could offer one explanation for the observed change in the basal expression level.

**Table 2:** Genomic PCR primers

Gene (region)	Location	Primer sequences (5'-3')
*ACOX1 *(distal)	-4919 to -4643	TGAGCTCTT**GATCTCCTCCTCAGAGTCATAG** GAGTCTAGA**CTGGCAATCTTAGCAGAGTTC**
*ACOX1 *(published)	-1646 to -1374	TGAGCTCTT**GAACTAGAAGGTCAGCTGTC** GGGTCTAGA**CTAGCCTGTCTGTAGTCTGTG**
*ACOX1 *(intron 1)	+599 to +716	TGAGCTCTT**GTGATTCAGGGAGGGTGGAAC** GGGTCTAGA**CTGGCTGCGAGTGAGGAAG**
*ACOX1 *(intron 2)	+2822 to +3154	TGAGCTCTT**GAGATAGAGTAACTCCTCCTAG** GAGTCTAGA**GAAGTGTGTCAAAGGGTGTG**
*ANGPTL4 *(distal)	-6765 to -6535	TGAGCTCTT**GAACTAGAAGGTCAGCTGTC** GAGTCTAGA**ATACACTCATGCAGGGTGAGG**
*ANGPTL4 *(cluster)	+2829 to +3610	TGAGCTCTT**CTCCGTTCATCTCGAACCAC** GAGTCTAGA**CATCTCAGAGGCTCTGCCTG**
*APOC3 *(distal 1)	-6429 to -6143	TGAGCTCTT**GCTCAGGCGATAGTTAGAAG** GAGTCTAGA**CTGGATGGTCCCACTCCAGTG**
*APOC3 *(distal 2)	-4249 to -3886	TGAGCTCTT**GACTATGAGGTGACATCCAGG** GAGTCTAGA**GGACACACAGGCAGTACGTG**
*APOC3 *(proximal)	-870 to -568	TGAGCTCTT**GGCAGTGAGGGCTGCTCTTC** GGGTCTAGA**CATCTCTGGGTTTCAATCCAG**
*APOC3 *(published)	-262 to -3	ATTTCTAGA**CAGTCAGCTAGGAAGGAATGAG** GGGTCTAGA**CTAGGGATGAACTGAGCAGAC**
*APOC3 *(intron)	+2424 to +2722	TGAGCTCTT**GATCACACAACTAATCAATCCTC** GAGTCTAGA**CTCAACTTCACTGGACGACAG**
*APOA1 *(proximal)	+7701 to +8022 (relative to *APOC3 *TSS)	TGAGCTCTT**CCTTCTCGCAGTCTCTAAGC** GAGTCTAGA**GCCAACACAATGGACAATGG**
*CPTIB*	-306 to -64	ATTTCTAGA**CAGAGTCTCGTGAGGATGGTG** GGGTCTAGA**GTTAGCGTTCATGCTGCCAG**
*PPARa *(distal)	-1376 to -1156	TGAGCTCTT**CTGGCTAACATGTGCAAGAG** GGGTCTAGA**CACTGTGCTATTTGTGGCAG**
*PPARa*(proximal)	-938 to -634	TGAGCTCTT**CTCCTTGCTCTGGCAGAGTC** GGGTCTAGA**CTCAGAAGTGCGTAGGGTG**
*RVRa*(distal)	-7279 to -7040	TGAGCTCTT**GACCTTCCCAAGCCAAGAAC** GAGTCTAGA**CACTAACCTCACAGACCACTG**
*RVRa*(proximal)	-510 to -70	TGAGCTCTT**CTGGAGGTGTTCTCCCTAAG** GTGTCTAGA**CTGCGCAACGACAAGACTG**
*RVRa*(TSS)	-510 to +119 (subcloned -266 to +119)	TGAGCTCTT**CTGGAGGTGTTCTCCCTAAG** GTGTCTAGA**TTTCACTCTGCCAATCTCAGC**
*SULT2A1*	-6104 to -5797	ATTTCTAGA**CTTGAATGGAAATGCCTGCTC** GGGTCTAGA**GACTGGGAAGTGGGAGGAGT**
*UCP3 *(distal)	-9680 to -9349	TGAGCTCTT**CTCTAGTCTAAGTGCCTTGTC** GAGTCTAGA**GTAACAGTGAGCCTCTGGTCTG**
*UCP3 *(proximal)	-396 to -89	TGAGCTCTT**GTACCTATCTCATAGGATTGTG** GTGTCTAGA**GTTGACAGCCTGATCACTTGAC**
*UCP3 *(cluster)	+2036 to +2303	TGAGCTCTT**CAGGACTATGGTTGGACTGAAG** GGGTCTAGA**GATGGGAGGAGGCAAGGAAG**
*UCP3 *(intron)	+5971 to +6236	TGAGCTCTT**CTCGTGCTGAGCACTTTACAC** GAGTCTAGA**CACTTGTTGGGTCCATTCTAAC**
*LASS1 *(region 1)	-5297 to -4917	TGAGCTCTT**CTGATGTGCAATCTCAGACAG** GAGTCTAGA**CTCAGTCTCCACCATGAAGG**
*LASS1 *(region 2)	-2819 to -2499	TGAGCTCTT**CCTCCCAGATGTCACCATTG** GAGTCTAGA**CCTCTTTTGCCACTTCCCTC**
*LASS1 *(region 3)	-1389 to -978	TGAGCTCTT**GTGGAACAGGAGCCATAGAG** GGGTCTAGA**CATCGAGGAAGACACTGGTC**

Sequence and location of the primer pairs used for real-time PCR quantification of genomic regions containing putative REs within the nine PPAR target genes. The positions indicated are in relation to the respective annotated gene TSS. The same primers were used for subcloning; the gene-specific sequences are indicated in bold.

Of the nine upstream regions, the region of the *SULT2A1 *gene was shown to be the most active (Figure 4b,e). In both cell lines over-expression of PPARs clearly increased this fragment's basal activity as well as significant inducibility by all three PPAR ligands. A similar observation was made in HepG2 cells for the distal region of the *UCP3 *gene, an effect that was far more modest in HEK293 cells. In contrast, neither the distal regions of the genes *ACOX1 *and *RVRα *nor the region containing the published PPRE of the gene *ACOX1 *displayed any inducibility by PPAR ligands in either of the two cells lines. Therefore, they can be considered as negative controls. In addition, the distal regions of the genes *ANGPTL4 *and *APOC3 *were only inducible in HEK293 cells, whereas the *PPARα *gene's putative PPRE-containing region responded only in HepG2 cells to GW501516 treatment. Interestingly, in HEK293 cells, the distal regions of the genes *ANGPLT4*, *APOC3 *and *PPARα *showed the already described effects of increased basal activity with endogenous activators and subsequent suppression of the activity by PPAR subtype over-expression.

Of the five downstream regions, the intron 2 region of the *ACOX1 *gene and the cluster region of the *ANGPTL4 *gene (containing four putative PPREs) displayed a clear response to all three PPAR ligands in both cell lines. In contrast, the inducibility of the intronic region of the *APOC3 *gene was far more modest (Figure 4c,f). Individual mutagenesis of the *ANGPLT4 *REs was carried out and this resulted in reduced activity, thus demonstrating that the other REs, in addition to the published PPRE, contribute to the responsiveness of this region (data not shown). Finally, the cluster and intronic region of the *UCP3 *gene responded only in HEK293 cells to GW501516 treatment.

In summary, of the 23 investigated genomic regions containing putative PPREs, up to 17 display significant inducibility in the presence of PPAR ligands (Table 3).

**Table 3:** Functionality of genomic regions

Genomic region	Predicted binding	Response in RGA	Association of PPARα	Association of RXRα	Association of pPol II	PPRE status
*ACOX1 *intron 1	Strong	+	+	+	+	+
*APOC3 *proximal	Weak	Down	+	+	+	+
*APOC3 *published	Medium	Down	+	+	-	+
*APOA1 *proximal	Weak	+/down	+	+	+	+
*CPT1B*	Strong	+	-	-	-	±
*PPARα *proximal	Medium	+	+	+	+	+
*RVRα *proximal	Medium	+	+	+	+	+
*RVRα *TSS	No DR1	-	+*	+*	+*	-
*UCP3 *proximal	Medium	Down	+	+	+	
*ACOX1 *distal	Medium	-	-	-	-	-
*ACOX1 *published	Not binding	-	-	-	-	-
*ANGPLT4 *distal	Weak	+/down	+	+	-	+
*APOC3 *distal 1	Medium	±	-	-	-	-
*APOC3 *distal 2	Medium	±	-	-	-	-
*PPARα *distal	Strong	+/down	+	+	+	+
*RVRα *distal	Weak	-	+	+	-	±
*SULT2A1*	Strong	+	+	+	+	+
*UCP3 *distal	Strong	+	+	+	+	+
*ACOX1 *intron 2	Medium	+	+	+	+	+
*ANGPTL4 *cluster	Strong	+	-	+	+	+
*APOC3 *intron	Strong	+	-	-	-	±
*UCP3 *cluster	Weak	-	-	-	-	-
*UCP3 *intron	Medium	Down	+	+	+	+

The data from reporter gene assay (RGA) and ChIP assays are summarized for each genomic region tested. The PPRE status indicates the conclusion drawn from the assays concerning the functionality of each region, with '+' assigned to functional regions, '-' to non-functional regions and '±' where the two assays were not in agreement. *Impossible to assess independent of adjacent region.

### Association of PPARs and their partner proteins to PPRE-containing regions

**Figure 5:**
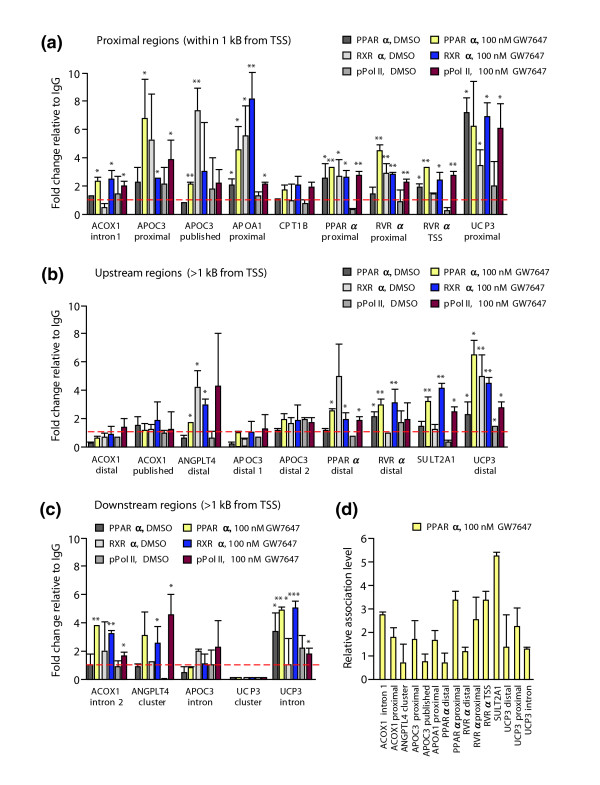
Association of genomic regions of PPAR target genes with PPARs and their partner proteins. Chromatin was extracted from HEK293 cells that had been treated with solvent (DMSO) or for 120 minutes with 100 nM GW7647. The association of PPARα, RXRα and pPol II was monitored by ChIP assays with respective antibodies on genomic regions of the eight PPAR target genes that are **(a) **close to the TSS, **(b) **upstream of the TSS and **(c) **downstream of the TSS; for location see Figure 3 and Table 2. Since the *APOC3 *gene is not expressed in HEK293 cells, the data for its four genomic regions were obtained using chromatin derived from HepG2 cells. Real-time quantitative PCR was performed on chromatin templates and the fold change of the antibody-precipitated template in relation to an IgG-precipitated specificity control template was calculated. **(d) **PPARα shows specific association with 15 of the 23 tested regions and the relative association with these regions is shown. Columns represent means of at least three experiments and bars indicate standard deviations. Two-tailed Student's *t*-tests were performed to determine the significance of association in reference to IgG controls (*p < 0.05, **p < 0.01, ***p < 0.001).

The same 23 genomic regions of the eight PPAR target genes were investigated by chromatin immuno-precipitation (ChIP) assays with chromatin extracts from HEK293 cells (or from HepG2 cells for regions from the *APOC3 *gene) that were treated with solvent or for 120 minutes with the PPARα ligand GW7647 (Figure 5). We assessed these regions for the binding of PPARα, its partner receptor RXRα and pPol II (the latter as a sign for a direct connection between the RE-containing region and the TSS). Chromatin templates were analyzed by quantitative real-time PCR and the specificity of the antibodies for the three proteins was compared with the non-specific background binding to IgG. Of the 23 tested regions, the region of the *CPT1B *gene, the distal and published region of the *ACOX1 *gene, the distal 1, distal 2 and intronic region of the *APOC3 *gene and the cluster of the *UCP3 *gene did not show specific binding of any of the three proteins. For the two regions of the *ACOX1 *gene this result confirmed their failure in the previous functionality test (Figure 4). The 16 other regions showed a significant association with PPARα in the presence of ligand. When comparing the relative association levels of PPARα under these conditions, we found that the most prominent binding was to the region of the *SULT2A1 *gene, followed by the regions of the *RVRα *TSS and the proximal region of the *PPARα *gene (Figure 5d). Interestingly, the latter two regions as well as the proximal regions of the genes *APOA1 *and *UCP3*, the distal region of the *RVRα *gene and the distal and intronic region of the *UCP3 *gene even displayed ligand-independent binding of PPARα. Similarly, a GW7647-independent association of RXRα was found on the published region of the *APOC3 *gene, on the proximal regions of the genes *APOA1*, *PPARα *and *UCP3 *and on the distal regions of the genes *ANGPTL4 *and *UCP3*. In contrast, no statistically significant binding of pPol II, irrespective of the presence of ligand, was found on the published region of the *APOC3 *gene and in the distal regions of the genes *ANGPTL4 *and *RVRα*.

Taken together, PPARα and RXRα associate in living cells with 16 of the 23 genomic regions. Thirteen of these regions also associate with pPol II, twelve of which show functionality in reporter gene assays (Figure 4, Table 3). With the exception of the *CPT1B *gene, the tested PPAR target genes possess one to three of these tested regions. The regions show neither positional bias nor do they preferentially contain evolutionarily conserved PPREs. However, each of them contains at least one predicted medium or strong PPRE. Three of the twelve fully functional regions, the *ANGPTL4 *cluster region, the proximal region of the *PPARα *gene and the distal region of the *SULT2A1 *gene, were already known, but we identified two alternative regulatory regions for each of the genes *ACOX1 *and *APOC3*, and one for the *RVRα *gene. We also found one additional regulatory region for the *PPARα *gene. Finally, for the *UCP3 *gene, for which no regulatory regions had so far been described to account for the effect of PPAR ligands on its mRNA transcription, we identified three functional areas.

### Clustering of PPAR target genes by self-organizing maps

**Figure 6:**
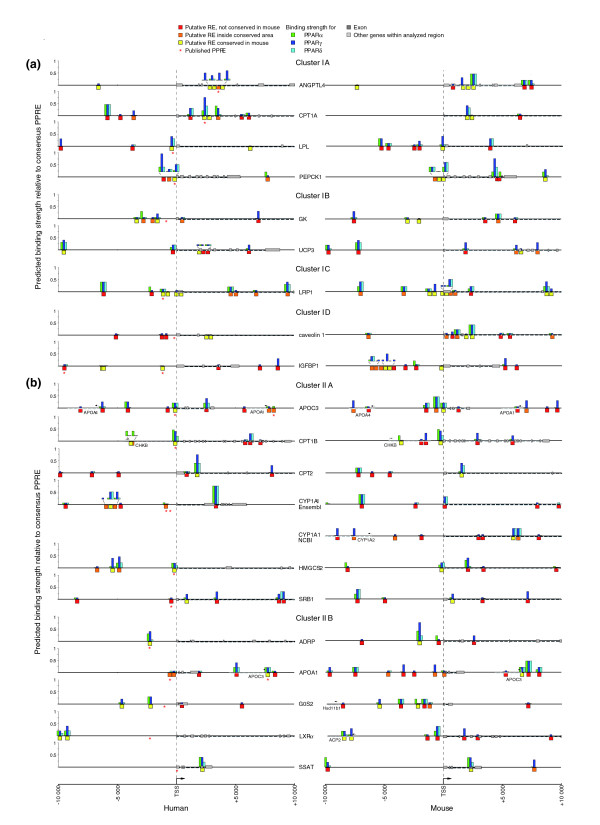
SOM analysis of established primary PPAR target genes, clusters I and II. Overview of the genomic organization of 38 known human PPAR target genes (left) and their mouse orthologs (right); 10 kB upstream and downstream of the TSS are shown in this and Figure 7. Please note that for the mouse *CYP1A1 *gene and the human *FADS2 *gene, there are discrepancies between the Ensembl (E) and NCBI (N) databases; therefore, both versions are shown. Putative PPREs (red boxes, no conservation; orange boxes, within conserved area; yellow boxes, conserved) were identified by *in silico *screening of the genomic sequences and are classified according to their degree of conservation between mouse and human. Already published PPREs are indicated by an asterisk. For each of the predicted PPREs, the calculated binding strengths of PPARα (green), PPARγ (dark blue) and PPARβ/δ (light blue) in reference to a consensus DR1-type PPRE are represented by column height. All putative PPRE sequences are available on request. The genes were sorted by SOM analysis with respect to overall PPRE pattern similarity and their evolutionary conservation into **(a) **cluster I and **(b) **cluster II.

**Figure 7:**
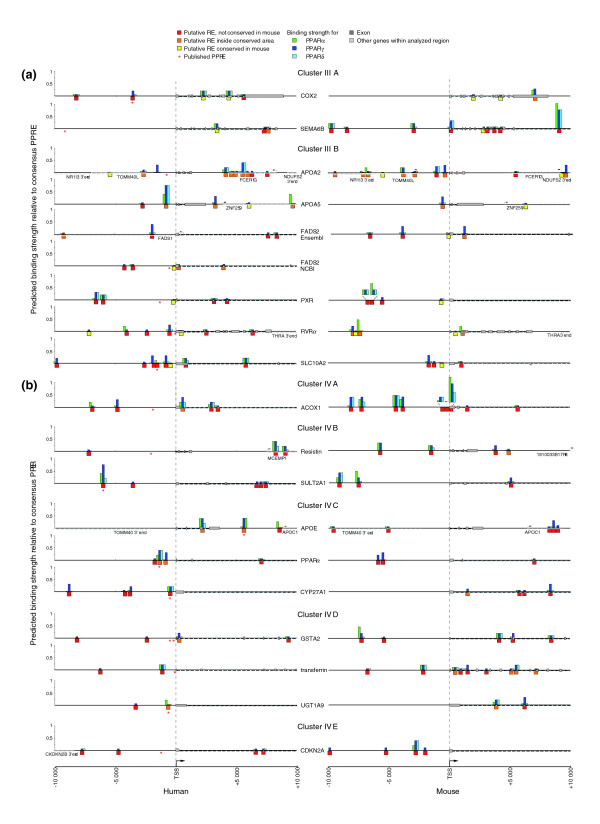
SOM analysis of established primary PPAR target genes, clusters III and IV. The genes were sorted by SOM analysis with respect to overall PPRE pattern similarity and their evolutionary conservation into **(a) **cluster III and **(b) **cluster IV. For more details, see the Figure 6 legend.

The common feature of the eight investigated PPAR target genes appears to be a prevalence for strong PPREs at a distance of up to 10 kB from the TSS. With the aim of extending this conclusion, we next compared all human genes that are known as primary PPAR targets. The genes were selected according to the following criteria: mRNA or protein level changes were reported for human cells or tissues; a PPRE was described for the human gene and it was experimentally confirmed by either gelshift, reporter gene or ChIP assay. This resulted in 30 additional genes, for which we performed *in silico *analysis for putative REs up to a distance of 10 kB from their respective TSSs (as done for the first eight genes; see Figure 3). In addition, for all of the 38 genes, the orthologous mouse gene was investigated in the same way (Figures 6 and 7). From these data, overview figures for each gene were constructed that show the location of the PPREs (x-axis) and their respective predicted binding strength (y-axis). In order to reveal further characteristics of the genes and their PPREs, such as overall similarity of their patterns and evolutionary conservation, they were clustered using a self-organizing map (SOM) algorithm followed by Sammon's mapping to illustrate the clusters. The input dataset of the SOM consisted of six variables, which are the sum of the predicted binding strength (BS), the number of conserved strong and medium binding sites (CS) and the number of weak binding sites (CW) both for the human and the mouse gene (Additional data file 5). An initial map resulted in four clusters, which were then each separated in the final map in up to five subclusters (Figures 6 and 7).

While the evolution of protein-coding sequences within genomes is well understood, the same cannot be said of the regions regulating transcription. The conservative model, often utilized as a limit for *in silico *binding site predictions, requires the strict preservation of binding site sequence and location. However, recent research on enhancer evolution has challenged this view. From these data, it appears possible to maintain overall functional conservation of regulatory elements over long evolutionary times, despite having high structural architecture turnover rates [16]. This feature has not been widely explored with human target genes, but is important to consider for target gene prediction. We therefore sought to identify traces of binding site evolutionary patterns in the clusters of this dataset. In general, clusters I and II contain genes that are well conserved between human and mouse, while the genes in cluster III are more divergent and those in cluster IV show no evolutionary conservation of PPREs.

Cluster I contains genes that carry multiple conserved PPREs (Figure 6a). Cluster IA contains the genes *ANGLPTL4*, *CPT1A *[29], *lipoprotein lipase *(*LPL*) [30] and *phosphoenolpyruvate carboxykinase *(*PEPCK*) 1 [31], which have, in both human and mouse, multiple conserved regions with strong REs. While each gene contains a conserved medium/strong element, a subset of genes expanded this set: human *CPT1A *and mouse *LPL *show significant enrichment of *de novo *binding sites compared to their orthologs. Cluster IB contains the genes *glycerol kinase *(*GK*) [32] and *UCP3*, which have multiple conserved RE regions. The distal UCP3 PPRE is conserved as a strong PPRE but is outside the 10 kB window in mouse. The cluster of PPREs in the human *GK *promoter seems to have lost significance in the mouse. The *lipoprotein receptor-related protein 1 *(*LRP1*) [33] gene represents cluster IC, in which multiple conserved REs are strong in mouse but weaker in human. Together with the genes *GK *and *LRP1*, the genes found in cluster ID, *caveolin 1 *[34] and *insulin-like growth factor binding protein 1 *(*IGFBP1*) [18], exhibit a retain-loss pattern concerning conserved PPREs, where only one species retained a cluster of strong PPREs. In the case of the *LRP1 *gene, this appears to have arisen in the context of several compensating *de novo *binding sites.

Cluster II differs from cluster I by having one or two strong or medium conserved REs in human, which are found in a comparable strength and location in mouse (Figure 6b). This cluster is subdivided into two clusters. Cluster IIA contains the genes *APOC3*, *CPT1B*, *CPT2 *[35], *cytochrome P450 *(*CYP*) *1A1 *[36], *3-hydroxy-3-methylglutaryl-CoA synthase 2 *(*HMGCS2*) [37] and *scavenger receptor B1 *(*SRB1*) [38], which have relatively comparable PPRE content. In contrast, cluster IIB contains the genes *adipose differentiation-related protein *(*ADRP*) [39], APOA1, *G0/G1 switch gene 2 *(*G0S2*) [40], *liver X receptor *(*LXR*)*α *[41] and *spermidine/spermine N1-acetyltransferase *(*SSAT*) [42], which exhibit an increase in PPRE content in the mouse gene.

Cluster IIIA, which contains the genes *cyclooxygenase 2 *(*COX2*) [43] and *semaphorin *(*SEMA*) *6B *[44], extends the pattern observed above with an opposite trend; the human ortholog contains one or two medium/strong REs, which are conserved but only weak in the mouse (Figure 7a). Possible compensating elements appeared in the mouse *SEMA6B *gene, while this is not the case for the mouse *COX2 *gene. Also, cluster IIIB contains one or two conserved REs, but they are weak in both human and mouse. This cluster comprises the genes *APOA2 *[45], *APOA5 *[46], *fatty acid desaturase 1 *(*FADS2*) [47], *pregnane X receptor *(*PXR*) [48], *RVRα *and *solute carrier *(*SLC*) *10A2 *[49]. Interestingly, these genes each have novel binding sites at nearly similar locations.

Cluster IV contains genes that carry one or more REs, but none of them is conserved (Figure 7b). The *ACOX1 *gene represents cluster IVA, in which multiple strong, but non-conserved, REs are found in both species. The genes *resistin *[50] and *SULT2A1 *form cluster IVB; they have one or two strong non-conserved REs in human and multiple REs in mouse. The genes *APOE *[51] and *PPARα *are in cluster IVC, which is characterized by one strong RE in the mouse ortholog and one or more non-conserved REs in the human gene. In cluster IVD are the genes *CYP27A1 *[52], *glutathione S-transferase *(*GST*) *A2 *[53], *transferrin *[54] and *UDP-glycosyltransferase *(*UGT*) *1A9 *[55], which carry one or two medium, non-conserved REs in both species. Finally, the *cyclin-dependent kinase inhibitor 2A *(*CDKN2A*) [56] gene represents cluster IVE, in which strong or medium non-conserved REs are found in mouse and but only weak REs are found in human.

In summary, SOM clustering of the 38 presently known human PPAR target genes sorts them into four clusters, of which the first three contain different numbers of evolutionarily conserved REs, while the 10 genes of cluster IV are characterized by having non-conserved REs. Interestingly, although for some genes a conservation of the PPRE pattern is evident, significant diversity in the composition of PPREs is visible as well.

### Evolutionary preservation patterns of PPREs in the genes *ACOX1 *and *ANGPLT4*

**Figure 8:**
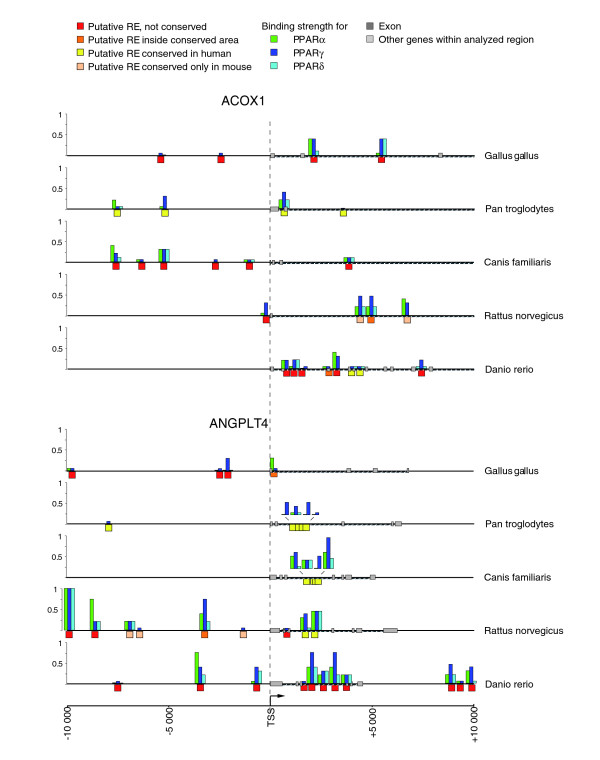
Conservation patterns across multiple species. The genes *ACOX1 *and *ANGPLT4 *from chicken, chimpanzee, dog, rat and zebrafish were also analyzed. Putative PPREs (red boxes, no conservation; orange boxes, within conserved area; yellow boxes, conserved in human; pink boxes, conserved in mouse) were identified by *in silico *screening of the genomic sequences. For each of the predicted PPREs, the calculated binding strengths of PPARα (green), PPARγ (dark blue) and PPARβ/δ (light blue) in reference to a consensus DR1-type PPRE are represented by column height. All putative PPRE sequences are available on request.

In order to explore the evolutionary preservation patterns of PPREs further, the genes *ACOX1 *and *ANGPLT4 *from the genomes of chicken, chimpanzee, dog, rat and zebrafish were also analyzed (Figure 8). In the genome of the chimpanzee (*Pan troglodytes*), the closest relative to human, four conserved PPREs were located in the *ACOX1 *gene, but the intron 2 region is missing. The respective human PPRE is not conserved in any of the species analyzed, suggesting that it is human-specific. Mouse and rat (*Rattus norvegicus*) share two PPREs, although both also contain a unique set of further REs. The analysis of the *ACOX1 *gene in chicken (*Gallus gallus*), dog (*Canis familiaris*) and zebrafish (*Danio rerio*) is in accordance with the overall pattern of relatively species-specific composition of PPREs. Within mammals, the cluster of intronic REs of the *ANGPLT4 *gene is rather well conserved. The closest PPRE pattern in comparison to the human gene is observed in the dog. Two intronic PPREs are also present in rat, but a significant expansion in PPREs seems to have occurred in this species, including a distal consensus RE. The zebrafish *ANGPLT4 *gene is also profoundly enriched with strong PPREs, whereas the chicken gene has a quite poor PPRE content. Moreover, the loss of the published PPRE is observed in the chimpanzee gene. Therefore, while this gene is an example of a much more preserved PPRE pattern, significant diversification is evident amongst the genomes analyzed and not all functional PPREs are conserved.

In conclusion, the SOM analysis and further genome comparisons together suggest that functional RE choice is evolutionarily flexible with respect to first gaining and then maintaining responsiveness to PPARs. In effect, integration of the stabilizing selection model into target gene identification and characterization may more faithfully identify PPAR targets.

### Identifying PPAR target genes in human chromosome 19

For the purpose of target gene identification, the SOM analysis (Figures 6 and 7) indicated that either the presence of at least one strong PPRE or more than two medium PPREs within the 20 kB surrounding the annotated TSS of a gene is a strong indication for a PPAR target gene. In this way, 28 out of the 38 human genes (74%) would have been identified as PPAR targets. Similarly, for 29 of these 38 genes (76%) the analysis of their murine ortholog would have come to the same conclusion. A combination of these two criteria (passing the threshold in either the human or mouse ortholog) would have identified 37 of the 38 genes (97%) as PPAR targets. *UGT1A9 *could be detected from the list of genes having two medium REs. While location clearly is not a major determinant of PPRE functionality, the screening of these regions in 500 bp windows indicates modest enrichment in the proximal promoter (24% of genes have a medium/strong PPRE close to the TSS; data not shown).

To explore this concept further, we selected the gene-dense human chromosome 19 (63.8 MB, 1,445 known genes in Ensembl, release 41) and its syntenic mouse regions (956 genes have known orthologs) and screened both species for medium and strong PPREs (based on a PPARγ prediction). We extracted from four human microarray datasets [8,57-59] those genes located on chromosome 19 that were shown to be regulated and determined whether these passed the criteria derived from the 38 target genes (that is, having a strong PPRE, a proximal medium PPRE or more than two medium PPREs). Typically, each dataset contained 6 to 8 genes from chromosome 19, out of which at least 5 (71-100%) passed the criteria in either or both species, and typically 1 or 2 genes had 2 medium PPREs only. This implies that the sensitivity of detecting targets based on our screen for medium and strong PPREs is high.

The background frequency of strong PPREs calculated by the total number of predicted strong PPREs divided by the length of chromosome 19 was found to be 0.66 for the 20 kB region considered for each gene, whereas that of medium or strong PPREs is 1.3. Twenty percent of genes of chromosome 19 contain a co-localizing strong PPRE and an additional four percent have more than two medium PPREs or a proximal medium PPRE. These numbers suggest a total of 4,000 to 5,000 targets for PPARs in the human genome, if no false positives are assumed. A complete evaluation of the selectivity is complicated by the restricted expression profiles of the predicted genes, which prevents simple read-outs from individual target tissues (regulation of only two genes from chromosome 19 was detected on more than two microarrays used earlier). We chose a focused list for further inspection, by requiring detection in human and mouse. In total, 116 genes (12.1%) from chromosome 19 pass the filter (Table 4). Furthermore, all 1,445 human genes were screened for high enrichment of PPREs, that is, for assemblies of at least three medium or strong PPREs (of which at least one was strong). These even more stringent criteria were fulfilled by 50 genes of both species (bold entries in Table 4) and an additional 75 human genes (Additional data file 6), a sample that represented 8.7% of all genes from human chromosome 19.

**Table 4:** Predicted PPAR target genes in human chromosome 19

Ensembl ID (human)	Gene name	Ensembl ID (mouse)
ENSG00000004776	***Heat-shock protein beta-6***	ENSMUSG00000036854
ENSG00000004777	***TC10/CDC42 GTPase-activating protein***	ENSMUSG00000036882
ENSG00000005007	*Regulator of nonsense transcripts 1*	ENSMUSG00000058301
ENSG00000010310	*Gastric inhibitory peptide receptor*	ENSMUSG00000030406
ENSG00000032444	*Neuropathy target esterase*	ENSMUSG00000004565
ENSG00000039987	*Bestrophin-2*	ENSMUSG00000052819
ENSG00000060566	*cAMP responsive element binding protein 3-like 3*	ENSMUSG00000035041
ENSG00000063176	***Sphingosine kinase 2***	ENSMUSG00000057342
ENSG00000063241	*Isochorismatase domain containing 2*	ENSMUSG00000052605
ENSG00000064547	***Lysophosphatidic acid receptor Edg-4***	ENSMUSG00000031861
ENSG00000072954	*Transmembrane protein 38A*	ENSMUSG00000031791
ENSG00000072958	*AP-1 complex subunit mu-1*	ENSMUSG00000003309
ENSG00000076944	*Syntaxin binding protein 2*	ENSMUSG00000004626
ENSG00000077348	*Exosome complex exonuclease RRP46*	ENSMUSG00000061286
ENSG00000079435	***Lipase, hormone sensitive***	ENSMUSG00000053714
ENSG00000080031	*Protein tyrosine phosphatase, receptor type, H precursor*	ENSMUSG00000035429
ENSG00000080511	***Retinol dehydrogenase 8***	ENSMUSG00000053773
ENSG00000083807	*SLC27A5*	ENSMUSG00000030382
ENSG00000083838	***Zinc finger protein 446***	ENSMUSG00000033961
ENSG00000089327	*FXYD domain-containing ion transport regulator 5 precursor*	ENSMUSG00000009687
ENSG00000089639	***GEM-interacting protein***	ENSMUSG00000036246
ENSG00000099203	*Transmembrane emp24 domain-containing protein 1 precursor*	ENSMUSG00000032180
ENSG00000099308	*Microtubule-associated serine/threonine-protein kinase 3*	ENSMUSG00000031833
ENSG00000099331	***Myosin-9B***	ENSMUSG00000004677
ENSG00000099617	*Ephrin-A2 precursor*	ENSMUSG00000003070
ENSG00000099622	***Cold-inducible RNA-binding protein***	ENSMUSG00000045193
ENSG00000099800	***TIMM13***	ENSMUSG00000020219
ENSG00000104826	***Lutropin β chain precursor***	ENSMUSG00000038194
ENSG00000104859	*Splicing factor arginine/serine rich 16*	ENSMUSG00000061028
ENSG00000104863	*LIN-7 homolog B*	ENSMUSG00000003872
ENSG00000104870	*IgG receptor FcRn large subunit p51 precursor*	ENSMUSG00000003420
ENSG00000104918	***Resistin***	ENSMUSG00000012705
ENSG00000104936	***Myotonin-protein kinase***	ENSMUSG00000030409
ENSG00000104946	***TBC1 domain family member 17***	ENSMUSG00000038520
ENSG00000104960	***Prostate tumor overexpressed gene 1***	ENSMUSG00000038502
ENSG00000104980	***Import inner membrane translocase subunit TIM44***	ENSMUSG00000002949
ENSG00000105066	***Flt3-interacting zinc finger protein 1***	ENSMUSG00000061374
ENSG00000105173	***G1/S-specific cyclin-E1***	ENSMUSG00000002068
ENSG00000105204	***Dual specificity tyrosine-phosphorylation-regulated kinase 1B***	ENSMUSG00000002409
ENSG00000105287	*Serine/threonine-protein kinase D2*	ENSMUSG00000041187
ENSG00000105289	*Tight junction protein ZO-3*	ENSMUSG00000034917
ENSG00000105364	*Mitochondrial 39S ribosomal protein L4*	ENSMUSG00000003299
ENSG00000105374	*Natural killer cell protein 7*	ENSMUSG00000004612
ENSG00000105379	***Electron transfer flavoprotein subunit β ***	ENSMUSG00000004610
ENSG00000105398	*SULT2A1*	ENSMUSG00000074375
ENSG00000105447	***Glutamate-rich WD repeat-containing protein 1***	ENSMUSG00000053801
ENSG00000105467	***Synaptogyrin-4***	ENSMUSG00000040231
ENSG00000105516	***D site-binding protein***	ENSMUSG00000059824
ENSG00000105552	*Branched-chain-amino-acid aminotransferase*	ENSMUSG00000030826
ENSG00000105664	*Cartilage oligomeric matrix protein precursor*	ENSMUSG00000031849
ENSG00000105701	*38 kDa FK506-binding protein homolog*	ENSMUSG00000019428
ENSG00000105707	*Serine protease hepsin*	ENSMUSG00000001249
ENSG00000108106	*Ubiquitin-conjugating enzyme E2 S*	ENSMUSG00000060860
ENSG00000118046	*Serine/threonine-protein kinase 11*	ENSMUSG00000003068
ENSG00000119574	***Zinc finger protein 499***	ENSMUSG00000049600
ENSG00000123154	*Mitogen-activated protein kinase organizer 1*	ENSMUSG00000005150
ENSG00000125910	*Sphingosine 1-phosphate receptor Edg-6*	ENSMUSG00000044199
ENSG00000125912	***Nicalin precursor***	ENSMUSG00000020238
ENSG00000126246	***Transmembrane protein 149***	ENSMUSG00000036826
ENSG00000126247	*Calpain small subunit 1*	ENSMUSG00000001794
ENSG00000127526	*SLC35E1*	ENSMUSG00000019731
ENSG00000129355	*Cyclin-dependent kinase 4 inhibitor D*	ENSMUSG00000066860
ENSG00000129451	*Kallikrein-10 precursor*	ENSMUSG00000030693
ENSG00000129455	*Kallikrein-9 precursor*	ENSMUSG00000047884
ENSG00000130165	*Transcription elongation factor 1 homolog*	ENSMUSG00000013822
ENSG00000130288	*NADH dehydrogenase 1 α subcomplex subunit 13*	ENSMUSG00000036199
ENSG00000130300	*Plasmalemma vesicle-associated protein*	ENSMUSG00000034845
ENSG00000130303	*Bone marrow stromal antigen 2*	ENSMUSG00000046718
ENSG00000130402	*α-actinin-4*	ENSMUSG00000054808
ENSG00000130520	***U6 snRNA-associated Sm-like protein LSm4***	ENSMUSG00000031848
ENSG00000130522	*Transcription factor jun-D*	ENSMUSG00000071076
ENSG00000130669	***PAK 4***	ENSMUSG00000030602
ENSG00000130687	*AlkB*, *alkylation repair homolog 6 isoform 2*	ENSMUSG00000042831
ENSG00000130755	*Glia maturation factor*, *gamma*	ENSMUSG00000060791
ENSG00000130818	*Zinc finger protein 426*	ENSMUSG00000059475
ENSG00000130881	***Low-density lipoprotein receptor-related protein 3 precursor***	ENSMUSG00000001802
ENSG00000131398	*Potassium voltage-gated channel subfamily C member 3*	ENSMUSG00000062785
ENSG00000132024	*Coiled-coil and C2 domain-containing protein 1A*	ENSMUSG00000036686
ENSG00000133246	*PML-RARα-regulated adaptor molecule 1*	ENSMUSG00000032739
ENSG00000141837	*Voltage-dependent P/Q-type calcium channel subunit α-1A*	ENSMUSG00000034656
ENSG00000142009	***Pyroglutamyl-peptidase I***	ENSMUSG00000056204
ENSG00000142290	*FXYD domain-containing ion transport regulator 7*	ENSMUSG00000036578
ENSG00000142513	***Testicular acid phosphatase isoform β precursor***	ENSMUSG00000012777
ENSG00000142538	*Tuberoinfundibular peptide of 39 residues precursor*	ENSMUSG00000038300
ENSG00000142539	*Spi-B transcription factor*	ENSMUSG00000008193
ENSG00000160113	***COUP-TFγ ***	ENSMUSG00000002393
ENSG00000160318	***Claudin domain containing 2***	ENSMUSG00000038973
ENSG00000160396	***Homeodomain-interacting protein kinase 4***	ENSMUSG00000040424
ENSG00000161249	*Dermokine isoform β*	ENSMUSG00000060962
ENSG00000161558	***Transmembrane protein 143***	ENSMUSG00000002781
ENSG00000161677	*Josephin-2*	ENSMUSG00000038695
ENSG00000167460	*Tropomyosin α-4 chain*	ENSMUSG00000031799
ENSG00000167470	*Midnolin*	ENSMUSG00000035621
ENSG00000167578	*Ras-related protein Rab-4B*	ENSMUSG00000053291
ENSG00000167754	***Kallikrein-5 precursor***	ENSMUSG00000074155
ENSG00000167757	***Kallikrein-11 precursor***	ENSMUSG00000067616
ENSG00000167772	***ANGPLT4***	ENSMUSG00000002289
ENSG00000167775	***CD320 antigen precursor***	ENSMUSG00000002308
ENSG00000168813	*Zinc finger protein 507*	ENSMUSG00000044452
ENSG00000171236	*Leucine-rich α-2-glycoprotein precursor*	ENSMUSG00000037095
ENSG00000171443	***Zinc finger protein 524***	ENSMUSG00000051184
ENSG00000171570	*Egl nine homolog 2*	ENSMUSG00000058709
ENSG00000174521	***Tetratricopeptide repeat domain 9B***	ENSMUSG00000007944
ENSG00000174562	***Kallikrein-15 precursor***	ENSMUSG00000055193
ENSG00000176531	***Pleckstrin homology-like domain family B member 3***	ENSMUSG00000061511
ENSG00000178093	*Testis-specific serine/threonine-protein kinase 6*	ENSMUSG00000047654
ENSG00000180448	***Minor histocompatibility antigen HA-1***	ENSMUSG00000035697
ENSG00000180739	*Sphingosine 1-phosphate receptor Edg-8*	ENSMUSG00000045087
ENSG00000185761	***Thrombospondin, type I, domain containing 6***	ENSMUSG00000043822
ENSG00000185800	***Dystrophia myotonica WD repeat-containing protein***	ENSMUSG00000030410
ENSG00000186474	***Kallikrein-12 precursor***	ENSMUSG00000044430
ENSG00000196867	*Zinc finger protein 28 homolog*	ENSMUSG00000062861
ENSG00000197050	*Zinc finger protein 420*	ENSMUSG00000058402
ENSG00000198356	*Arsenical pump-driving ATPase*	ENSMUSG00000052456
ENSG00000204673	***AKT1 substrate 1***	ENSMUSG00000011096
ENSG00000205155	***Gamma-secretase subunit PEN-2***	ENSMUSG00000036835

All 956 genes of human chromosome 19 that have known mouse orthologs were screened *in silico *for strong and medium PPREs within 10 kB upstream and downstream of the gene's annotated TSS. All putative PPRE sequences are available on request. The 116 genes that carry, in both species, a strong PPRE or two or more medium PPREs, or a medium PPRE within 500 bp upstream of the TSS are listed. The 50 genes that pass the even more stringent criterion of three PPREs, including one strong, are highlighted in bold.

Comparing these lists with published microarray-derived lists of target genes suggests interesting candidates in different physiological contexts of PPARs, where genes showing evidence of regulation have already been detected. PPARs play a prominent role in lipid metabolism and homeostasis. Genes detected from chromosome 19 represent diverse functions, such as liberation and transport of lipids (*hormone sensitive lipase *[58] and the fatty acid transporter genes *SLC27A5 *and *low density lipoprotein related receptor 3*), signaling molecules affecting lipid homeostasis (*resistin *and the transcription factor gene *CCAAT/enhancer binding protein *[58], which is known to regulate leptin expression [60]) and the generation of modified lipids that may have signaling roles (*CYP4F8 *functioning in ω-oxidation and *LASS1 *functioning in ceramide synthesis). Genes with a function related to mitochondrial energy metabolism include several mitochondrial translocases (the genomic neighbor of the *APOE *gene *TOMM40 *[58] and the genes *translocase of inner mitochondrial membrane *(*TIMM*) *13 *and *44*) and mitochondrial enzymes and complex subunits (*branched-chain amino acid transferase *[58] and *electron transfer flavoprotein subunit β*).

Of relevance to cancer, several cell cycle regulating genes were found, such as *G1/S-specific cyclin E *[61], *p19*^*INK4d *^[58], *prostate tumor overexpressed gene*, *serine protease hepsin *[57] and those encoding the serine/threonine kinases associated with cell cycle regulation, *p21-activated kinase 4 *(*PAK4*) and *homeodomain-interacting protein kinase 4*. In addition, the prostate tumor marker *kallikrein-3 *[62] and several other *kallikrein *gene family members were detected. Kallikreins represent one gene family that likely arose by duplications on chromosome 19; other such families include zinc finger proteins, of which several also passed the filter and many are detected on microarrays.

Other physiological roles that have been more recently studied in connection with PPARs include regulation of immune reactions and muscle target genes. A large group of predicted genes has functions in the immune system, such as the genes *killer cell immunoglobulin-like receptor 2DL4 *[8], *natural killer cell protein 7 *and *bone marrow stromal antigen 2*. Putative muscle targets include the genes *myotonic dystrophy protein kinase *and *tropomyosin α4-chain *[8]. Interestingly, in connection with effects on the circulatory system, regulation of the lysophosphatidic acid (LPA) receptor gene *Edg-4 *[58,63] has been detected, and here we predict two other family members, *Edg-6 *and *Edg-8*, to also be regulated. LPA leads to contraction of vessels, which is also achieved by renin protein. Interestingly, among the genes detected is *chicken ovalbumin upstream promoter transcription factor *(*COUP-TF*) *γ*, which codes for a NR that is known to regulate *renin *expression [64].

### Validation of PPAR target genes on human chromosome 19

**Figure 9:**
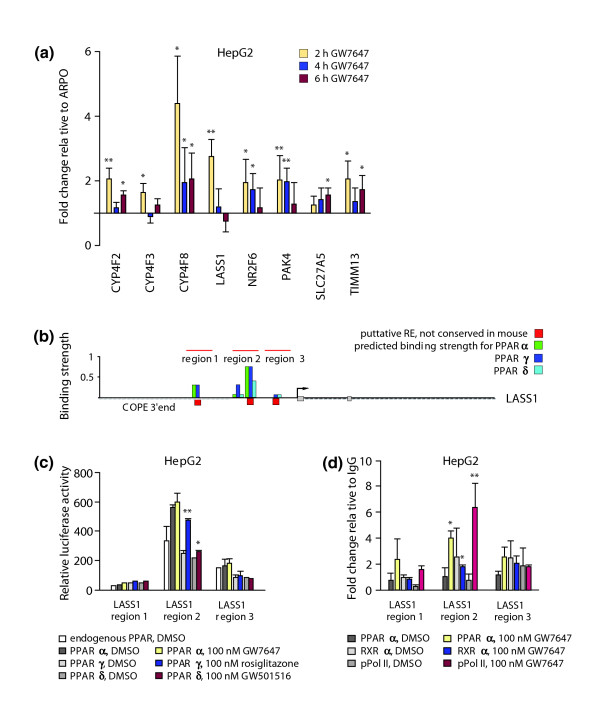
Validation of novel PPAR target genes on human chromosome 19. **(a) **Real-time quantitative PCR was used to determine the inducibility of the mRNA expression of the indicated eight PPAR target genes, relative to the control gene *RPLP0*, in HepG2 cells. The cells were stimulated for 2, 4 and 6 h with 100 nM GW7647. **(b) **An overview of the genomic organization of the human *LASS1 *gene; 10 kB upstream and downstream of the TSS are shown. Putative REs were identified by *in silico *screening and the calculated binding strengths of the PPAR subtypes are represented by columns in reference to a consensus DR1-type PPRE. All putative PPRE sequences are available on request. **(c) **Reporter gene assays were performed with extracts from HepG2 cells that were transiently transfected with *luciferase *reporter constructs containing genomic regions of the *LASS1 *gene together with empty expression vector (endogenous PPAR) or the indicated expression vectors for PPARα, PPARγ and PPARβ/δ. Cells were then treated for 16 h with solvent or PPAR subtype-specific ligands. Relative luciferase activity was determined and normalized to the activity of empty cloning vector control co-transfected with empty expression vector. **(d) **Chromatin was extracted from HepG2 cells that had been treated with solvent or for 120 minutes with 100 nM GW7647. The association of PPARα, RXRα and pPol II was monitored by ChIP assays with respective antibodies on three genomic regions of the *LASS1 *gene. Real-time quantitative PCR was performed on chromatin templates and fold change of antibody-precipitated template in relation to IgG-precipitated specificity control template was calculated. Columns in (a, c, d) represent means of at least three experiments and bars indicate standard deviations. Two-tailed Student's *t*-tests were performed to determine the significance (*p < 0.05, **p < 0.01).

From these lists, the six human genes *CYP4F8*, *LASS1*, *COUP-TFγ*, *PAK4*, *SLC27A5 *and *TIMM13 *were selected for real-time PCR evaluation of their response to the PPARα ligand GW7647 in HepG2 cells (Figure 9a). All these genes contain at least one strong and one medium PPRE predicted to bind PPARα. The genes *COUP-TFγ*, *PAK4 *and *TIMM13 *also showed enrichment in mouse (for *CYP4F8*, no 1:1 ortholog prediction exists). After 2 hours of treatment with ligand, all 6 genes showed a significant (between 1.8- and 4.2-fold) up-regulation of their mRNA (Figure 9a). For a more detailed analysis we selected the *LASS1 *gene. The *in silico *analysis of the gene suggested four non-conserved PPREs, of which the two strong REs in close vicinity (region 2) are the best candidates for forming the PPAR-responsive region of the *LASS1 *gene (Figure 9b). Functional analysis of three genomic regions in reporter gene assays in HepG2 cells indicated for region 2 a significant up-regulation by PPARγ and PPARβ/δ ligands and an even more prominent basal activity for the PPARα agonist (Figure 9c). The two other regions did not show a significant response to PPAR over-expression or ligand treatment. ChIP assays in HepG2 cells confirmed this result (Figure 9d). Treatment with GW7647 induced significant binding of PPARα, RXRα and pPol II to region 2, but not to regions 1 and 3. This suggests that the two strong PPREs in region 2 mediate the response of the *LASS1 *gene to PPAR ligands.

Taken together, the *in silico *screening of 956 genes from human chromosome 19 suggest that 12.1% of them have two or more PPREs in both the human and mouse orthologs; and 8.7% of all genes from chromosome 19 even carry an assembly of three or more PPREs. All six genes of this panel show early responses in their mRNA expression levels to the presence of a PPARα ligand. Using the *LASS1 *gene as an example, we demonstrated that the most likely region, containing two PPREs, is functional and recruits PPARα and RXRα and is associated with pPol II.

## Discussion

The identification of genes showing a primary response to PPARs and their ligands, the PPAR regulome, can be used for prediction of their therapeutic potential as well as their possible side effects. In this study, we present a method that incorporates both experimental- and informatics-derived evidence to arrive at a more reliable prediction of PPAR target genes. We provide here DNA-binding data for all three PPAR subtypes to a large panel of natural and synthetic DR1-type REs. We describe a PPRE classifier and test it together with two matrix methods based on the binding data to detect and predict the *in vitro *binding strength of PPAR-RXR heterodimers to their REs. Although all methods provide relatively good discrimination of sequences that bind PPARs from those that do not, the classifier also retained a close correlation between the prediction and the experimental binding strength and was chosen for the further assessment of PPAR target genes.

Previously, scoring of putative PPREs has been based on an alignment of a number of natural PPREs that were used to create PSWMs as opposed to the PSWM presented here, which uses experimentally verified strong or medium PPREs from our dataset. These are the basis for commonly used programs for *in silico *screening of NR REs, such as ConSite and JASPAR. However, longer binding sites, such as those of NRs composed of two half sites, could be especially challenging for weight matrix approaches because they create mathematical scores over the whole length of the binding sequence. Therefore, they may be prone to overcompensate for nucleotides that are totally unfavorable for binding at one position with scores from other positions.

Another assumption underlining the matrix scores is the base-independence assumption. This was recently challenged by a study that collected a large dataset of affinity data for the basic helix-loop-helix transcription factor family [65]. As the number of consensus variations was increased, predictions based on an affinity matrix that was created on the basis of single nucleotide variation data correlated poorly with experimental binding of multiple variation data. However, the utility of more complex models incorporating the base-dependence assumption has been challenged in the context of other transcription factors [66]. Although the data we present here seem to be in line with the observation that the correlation of matrix scores with binding strength is not obvious, systematic di- or trinucleotide screening data would be needed to challenge this idea. The usefulness of the matrix methods for the detection of binding sites is widely acknowledged and the two matrices presented here perform well in this regard.

Since our classifier performed well in all tests, it was chosen for binding strength prediction. As shown for the example of the REs of the human *UCP3 *gene, the *in silico *prediction of PPAR-RXR heterodimer binding strength fits well with their experimentally determined binding. Due to the large number of possible variations, we consider a ± 15% interval for a match between prediction and true binding strength as sufficient to evaluate the binding site composition of target genes. Moreover, the classifier is also sensitive, since optimal performance was already reached with a threshold for PPRE binding of 3% relative to the consensus.

We extended our *in silico *screening to a distance of 10 kB upstream and downstream of the TSS. This limit is above the restrictions of genome-wide promoter screens for transcription factor binding sites [15] and also acknowledges the rather recent understanding that regulatory sequences are symmetrically located around the TSS [67]. Our experimental analysis of 23 genomic regions of 8 validated PPAR target genes, which together contain 30 putative PPREs, indicated that the majority of them are functional (17 regions in HEK293 cells). Moreover, in living cells we detected for 12 regions a significant, mostly PPAR ligand-dependent association of PPARα, RXRα and pPol II. Prevalence of strong PPREs seems to be a common feature among direct PPAR target genes, since all eight investigated genes contain at least one functional region, each carrying at least one strong PPRE. Moreover, based on this set of functional regions, we could not detect any positional bias towards the TSS. This agrees with other data concerning the positioning of NRs relative to the TSS [15]. For example, for genes such as human *ACOX1 *and *RVRα*, where for historical reasons PPREs rather close to the TSS were reported, we show that the respective PPRE-containing regions were not functional. Instead, we suggest for these two genes (and also for a couple of others) a number of alternative PPAR-responding regions more distant from the TSS or downstream of it. For the *UCP3 *gene, for which no PPRE had so far been reported, we show three PPAR-responding regions containing strong and medium strength PPREs. In conclusion, our binding site strength prediction scheme allows us to identify novel, functional PPREs from known PPAR targets that are experimentally verifiable.

Meta-analysis of 38 established PPAR responding genes indicated that the most convincing PPAR targets contain two or more strong (or at least medium) PPREs in both the human and mouse orthologs. Our detailed knowledge of the DNA-binding preferences of PPARs combined with insights from the meta-analysis of a large number of PPAR targets gave us the advantage of being able to detect characteristics of target genes that were ignored before. We investigated genomic variables, such as PPRE location relative to the TSS and their evolutionary conservation, in an unbiased way. The identification of, in total, 13 subclusters in the set of established PPAR target genes suggests that evolutionary constraints to maintain responsiveness do not translate to the preservation of an identical PPRE pattern; instead, the appearance and fixation of novel sites adds flexibility. In effect, evolution has chosen a number of different strategies to acquire and maintain the responsiveness of genes to PPARs. Based on the set of known targets, this appears to manifest itself as an enrichment of strong binding sites. Furthermore, as suggested by our experimental evaluation of PPREs, more than one of these sites may be functional for any given time. By tracking this enrichment and turnover of binding sites from multiple species, our *in silico *screening approach has, compared to other methods, an increased chance to detect eventually all PPAR target genes in a chromosome or even a whole genome.

As an example, we screened human chromosome 19 for PPREs and predicted PPAR target genes. Among the 956 genes of human chromosome 19 for which we could identify mouse orthologs, we predicted 116 genes (12.1%) from both species to be PPAR target genes by tracking the appearance of strong sites, or enrichment of medium/strong sites in both species. This approach has the ability to detect targets with divergent PPRE composition. Such an analysis will be even be more powerful with the incorporation of additional genomes, in particular in the detection of PPAR targets that differ between human and mouse. By using only one species we limited our search to a more stringent screening for genes with both enrichment of PPREs (three or more PPREs) and a strong PPRE. This suggested that 118 of the 1,445 genes of human chromosome 19 (8.2%) are PPAR targets. These genes comprise interesting candidates representing physiological functions connected to PPAR. For a group of six genes that are representative for the predicted PPAR targets, all were experimentally proven to respond to PPAR ligands. This suggests that selectivity is also high when assessed in proper physiological contexts (tissues). We plan to extend our analysis beyond chromosome 19 to the whole human genome with the incorporation of more species as soon their complete sequences become available. Taking both whole chromosome 19 *in silico *screening trials together and extrapolating the results to the whole human genome, we suggest that approximately 10% of all human genes (an estimate of 2,000 to 2,500 genes) have the potential to be directly regulated by PPARs. Incidentally, this fits with experimental data regarding PPARα targets [13].

From the six representative genes of human chromosome 19 we selected the *LASS1 *gene for more detailed analysis. The *in silico *screening of this gene suggested that it has four REs in three regions. Of these, region 2 is the most obvious PPAR regulatory region, since it contains a strong PPRE in close vicinity to a medium PPRE. In fact, reporter gene and ChIP assays confirmed this prediction in reference to non-functional regions of the same gene. Together with the results observed with the *UCP3 *gene, this suggests that our method is a reliable approach not only to predict primary PPAR target genes on a genomic scale but also to identify *in silico *regulatory regions with functional PPREs for each individual gene.

## Conclusion

We present here the development of an experiment-based informatics method for more reliable prediction of PPAR target genes on the whole genome level and important insights into the relationship of different genomic variables to PPRE functionality and the turnover of their binding sites during evolution. This approach and the underlying concepts can also be applied to other members of the NR superfamily and explored for use with all DNA-binding transcription factors for which sufficient reliable DNA-binding data are available.

## Materials and methods

### *In silico *screening of putative PPREs using a PPRE classifier

Data for the *in vitro *binding of the three PPAR subtypes to 39 single nucleotide variations of a consensus PPRE [18] were sorted into classes I, II and III (Table 1). To address binding to multiple variations, a total of 136 DR1-type RE sequences were tested for the *in vitro *binding of PPAR-RXR heterodimers and then used to calculate the average binding strength of the three PPAR subtypes in each of the categories 1/0/0, 0/1/0, 2/0/0, 3/0/0, 1/1/0, 0/0/1, 0/2/0, 2/1/0, 1/0/1, 3/1/0 and 4/0/0 (Figure 1), where the numbers indicate the number of variations for the classes I, II and III, respectively. Other combinations resulted in less than 1% average binding (Additional data file 1) and were not considered for the PPRE search. Genomic sequences for human and mouse orthologous genes spanning a 10 kB distance to their respective TSSs were extracted from the Ensembl database (release 40, April 2006) and screened for DR1-type REs using in-house software named RESearch (source code and templates for searching for medium/strong PPREs are available upon request) [68]. A list of all possible PPREs belonging to medium and strong PPRE categories are described in template files that the program uses to annotate the sequence files. The naming of results corresponds to the category where the RE is found. The PPAR subtype-specific binding strength was predicted based on data from Figure 1. The conservation of the putative PPREs between human and mouse were evaluated using the Vertebrate Multiz Alignment and Conservation track available from the UCSC genome browser (NCBI releases for human and mouse genomes, hg18 and mm8, February 2006) [69]. The *ANGPLT4 *and *ACOX1 *genes were extracted from Ensembl for chicken (2.1, May 2006), chimpanzee (PanTro 2.1, March 2006), dog (CanFam 2.0, May 2005), rat (RGSC 3.4, November 2004) and zebrafish (Zv6, March 2006). Human chromosome 19 and its syntenic mouse regions were extracted from Ensembl release 41 and screened for putative PPREs of strong or medium predicted binding strength at a distance of up to 10 kB from each TSS. The Ensembl ortholog prediction was used to match the respective human and mouse genes.

### Construction of a PSWM and a PSAM

The PPARγ binding data were used to construct the matrices. For the weight matrix all medium and strong PPREs that contain multiple variations were included. This set of 20 sequences was used to calculate frequencies of each base-pair, which were then divided by the background frequency (assumed equal for all base-pairs). A pseudocount of 0.01 was introduced to the calculation to represent unobserved base-pairs. The values were converted to matrix weights by taking the natural logarithm of the corrected frequency values. The single nucleotide variation data were used to construct the affinity matrix. The binding strength of the different nucleotides (values between 0 and 1) in a given position was converted to a matrix value by setting each column sum equal to 1. To correlate matrix score with experimental binding strength, the equations of the lines fitted to the single nucleotide variation data (Additional data file 3) were used to convert a matrix score to a binding prediction.

### Comparison of *in silico *methods

Two sets of rules were used to define true positives (TPs), false positives (FPs), true negatives (TNs) and false negatives (FNs). To discriminate sequences that bind PPARs from those that do not, the following definitions were used: TP = the score is over the threshold and the sequence binds PPAR (in the case where the classifier score = average); TN = the score is below the threshold and the sequence does not bind PPAR; FP = the score is over the threshold but the sequence does not bind PPAR; FN = the score is below the threshold but the sequence binds PPAR. To compare if a prediction/score given by the method correlated with binding strength, the following definitions were used: TP = the prediction matches experimental binding with 15% of the consensus as the tolerance limit (5% for sequences predicted to bind less than 15%) and the sequence binds PPREs; TN = the observed binding is less than the prediction threshold (optimal thresholds were 3% for classifier, 30% or 0.0000015 for PSAM and 20% or 4.7 for the PSWM); FP = the observed binding is lower than predicted and outside the 15% tolerance interval for a match; FN = the observed binding is higher than predicted and outside the 15% tolerance interval for a match. These values were used to calculate the true positive and false positive rates: TPR = TP/P = TP/(TP + FN); FPR = FP/N = FP/(FP + TN).

The performance of the methods was compared by calculating predictions for the experimental data from Figure 1 and Additional data file 1 using the different methods. This dataset contains a well-defined true negative set (all non-binding sequences representing approximately 30% of data) and a well-defined true positive set.

### Cell culture

The human embryonal kidney cell line HEK293 and the human hepatocarcinoma cell line HepG2 were cultured in Dulbecco's modified Eagle's medium (DMEM) containing 10% fetal bovine serum (FBS), 2 mM L-glutamine, 0.1 mg/ml streptomycin and 100 units/ml penicillin in a humidified 95% air/5% CO_2 _incubator. Before use, the FBS was stripped of lipophilic compounds, such as endogenous NR ligands, by stirring it with 5% activated charcoal (Sigma-Aldrich, St Louis, MO, USA) for 3 h at room temperature. Charcoal was then removed by centrifugation and sterile filtration. Prior to mRNA or chromatin extraction, cells were grown overnight in phenol red-free DMEM supplemented with 5% charcoal-stripped FBS to reach a density of 50-60% confluency. Cells were then treated with either solvent (DMSO, 0.1% final concentration) or 100 nM of the PPARα agonist GW7647 (2-(4-(2-(1-cyclohexanebutyl-3-cyclohexylureido)ethyl)phenylthio)-2-methylpropionic acid), 100 nM of the PPARγ agonist rosiglitazone(5-((4-(2-(methyl-2-pyridinylamino) ethoxy)phenyl)methyl)-2,4-thiazolidinedione) or 100 nM of the PPARβ/δ agonist GW501516 (2-methyl-4-((4-methyl-2-(4-trifluoromethylphenyl)-1,3-thiazol-5-yl)-methylsulfanyl)phenoxy-acetic acid). GW7647 and GW501516 were purchased from Alexis Biochemicals (San Diego, CA, USA), while rosiglitazone was kindly provided by Dr Mogens Madsen (Leo Pharma, Ballerup, Denmark). The ligands were dissolved and diluted in DMSO.

### RNA extraction and quantitative real-time PCR

Total RNA was extracted using the Mini RNA Isolation II kit (ZymoResearch, HiSS Diagnostics, Freiburg, Germany). The RNA was purified and eluted according to the manufacturer's instructions (ZymoResearch). cDNA synthesis was performed for 1 h at 37°C using 1 μg of total RNA as a template, 100 pmol of oligo(dT_15_) primer and 40 units of reverse transcriptase (Fermentas, Vilnius, Lithuania) in a 40 μl volume. Subsequently, the cDNA was diluted 1:10 with H_2_O. Real-time quantitative PCR was performed in an IQcycler (BioRad, Hercules, CA, USA) using the dye SybrGreen I (Molecular Probes, Leiden, The Netherlands). Per reaction, 4 μl cDNA, 1 U FastStart Taq polymerase (Roche, Mannheim, Germany) and 2 mM MgCl_2 _were used. The PCR cycling conditions were: 45 cycles of 30 s at 95°C, 30 s at 60°C and 25 s at 72°C. The sequences of the gene-specific primer pairs for the PPAR target genes and the internal control gene *acidic riboprotein P0 *(*RPLP0*) are listed in Table 5. PCR product quality was monitored using post-PCR melt curve analysis. The fold inductions were calculated using 2^-(ΔΔCt)^, where ΔΔCt is the ΔCt_(PPAR ligand) _- ΔCt_(DMSO)_, ΔCt is Ct_(target gene) _- Ct_(*RPLP0*) _and Ct is the cycle at which the threshold is crossed.

**Table 5:** PCR primer pairs for quantitative real-time PCR

Gene	Primer pairs (5'-3')	Product size (bp)
*ACOX1*	GTATGGAATCAGTCAGAACGC CTTGTAAGATTCGTGGACCTC	261
*ANGPLT4*	GAGCCTCTCTGGAGGCTGGTG CAGTCGTGGTCTTCTTCTCTG	334
*APOC3*	CATGCAGGGTTACATGAAGCAC GTAGGAGAGCACTGAGAATAC	325
*CPT1B*	TTCTGCCTTTACTTGGTCTCCA GGGTCGAACATGCGGATCT	124
*PPARa*	TGCTGTCTTCTGTGATGAAC TCTGAGCACATGTACAATAC	268
*CYP4F8*	CATCTTCGCAATCCATCACAAC GACCACCTTCATCTCTGCCATC	174
*LASS1*	CAGCTTGAGTTCACCAAGCTC CACGATGTACAGGAACCAGTAG	266
*NR2F6*	GTGGCTTTCATGGACCAG CAGCATGTCTCTGATCAGTG	344
*PAK4*	GAGCGACTCGATCCTGCTGAC GACCAGATGTCTACCTCTG	173
*RVRa*	AGGACCAGACAGTGATGTTC CTTCTCGGAATGCATGTTGTTC	343
*SLC27A5*	CAGGTTGTGAGGGTAAGGTG CATCAGTTTGAACGTGCTGGTG	169
*SULT2A1*	GATTATGTAGTGGACAAAGCAC CAAGGAAGGGATCAGAGATG	296
*UCP3*	CACCTGCTCACTGACAACTTC GTTACGAACATCACCACGTTC	247
*TIMM13*	CAGAGGATGACGGACAAGTG GGTCACATGTTGGCTCGTTC	172
*RPLP0*	AGATGCAGCAGATCCGCAT GTGGTGATACCTAAAGCCTG	318

PCR product sizes are indicated.

### DNA constructs

Full-length cDNAs for human PPARα [70], human PPARγ [71], human PPARβ/δ [72] and human RXRα [73] were subcloned into the T7/SV40 promoter-driven pSG5 expression vector (Stratagene, La Jolla, CA, USA). The same constructs were used for both T7 RNA polymerase-driven *in vitro *transcription/translation of the respective cDNAs and for viral promoter-driven over-expression in mammalian cells. Selected genomic regions of PPAR target genes were cloned by PCR from human genomic DNA (for primers see Table 2) and fused with the *thymidine kinase *promoter driving the firefly *luciferase *reporter gene.

### Clustering of gene data using SOMs

PPAR target genes were clustered using Visual Data software (Visipoint OY, Kuopio, Finland), which is based on SOMs. These are artificial neural network algorithms in the unsupervised learning category that can visualize and interpret large high-dimensional datasets [74]. The map consists of a regular grid of processing units, so-called 'neurons', which are organized hierarchically in a pyramid-like fashion in several layers. Four adjacent neurons of the best matching unit form the neighborhood that gets updated. The lower levels of the map provide a coarse mapping of the data, while fine structures and clusters emerge when more neurons are used. For the best visualization, the SOM vectors were used as an input for the Sammon algorithm. The input dataset of the SOM consisted of six variables (shown according to final SOM clustering in Additional data file 5). The variables BS_H _and BS_M _represent the sum of predicted binding strength of n putative medium or strong PPREs (Σbs_n_, bs_i _= max{bs_PPARα_, bs_PPARγ_, bs_PPARβ/δ_}) found within the 20 kB of the analyzed region of each gene in human (H) and mouse (M), respectively. The remaining values indicate the number of conserved strong/medium (CS) or weak (CW) PPREs in human and mouse. Prior to SOM initialization the BS variables were scaled between 0 and 1 and the maximal resolution was set to 256. Finally, a Sammon's mapping algorithm (Visipoint OY) was applied to visualize the clustered groups in *n*-dimensional space in two dimensions. For this analysis the human and mouse sequences were treated as independent sequences. This may result in overestimation of conserved pairs; however, the evolutionary distance between the species is, in general, considered sufficient to offer useful information about conservation patterns.

### Gelshift assay

*In vitro *translated PPAR subtype and RXRα proteins were generated by coupled *in vitro *transcription/translation using their respective pSG5-based full-length cDNA expression constructs and rabbit reticulocyte lysate as recommended by the supplier (Promega, Madison, WI, USA). Protein batches were quantified by test-translations in the presence of [^35^S]-methionine. Gelshift assays were performed with 10 ng of the appropriate *in vitro *translated proteins. The proteins were incubated for 15 minutes in a total volume of 20 μl of binding buffer (150 mM KCl, 1 mM dithiothreitol, 25 ng/μl herring sperm DNA, 5% glycerol, 10 mM Hepes, pH 7.9). Constant amounts (1 ng) of [^32^P]-labeled double-stranded oligonucleotides (50,000 cpm) containing one copy of the respective REs were then added and incubation was continued for 20 minutes at room temperature. Protein-DNA complexes were resolved by electrophoresis through 8% non-denaturing polyacrylamide gels (mono- to bisacrylamide ratio 19:1) in 0.5 × TBE (45 mM Tris, 45 mM boric acid, 1 mM EDTA, pH 8.3) for 90 minutes at 200 V and quantified on a FLA-3000 reader (Fuji, Tokyo, Japan) using ScienceLab99 software (Fuji).

### ChIP assays

Nuclear proteins were cross-linked to genomic DNA by adding formaldehyde for 5 minutes directly to the medium to a final concentration of 1% at room temperature. Cross-linking was stopped by adding glycine to a final concentration of 0.125 M and incubating for 5 minutes at room temperature on a rocking platform. The medium was removed and the cells were washed twice with ice-cold phosphate-buffered saline (140 mM NaCl, 2.7 mM KCl, 1.5 mM KH_2_PO_4_, 8.1 mM Na_2_HPO_4_•2H_2_O). Cells were first collected by scraping into ice-cold phosphate-buffered saline (PBS). After centrifugation the cell pellets were resuspended in lysis buffer (1% SDS, 10 mM EDTA, protease inhibitors (Roche), 50 mM Tris-HCl, pH 8.1) and the lysates were sonicated to result in DNA fragments of 300 to 1,000 bp in length. Cellular debris was removed by centrifugation and the lysates were diluted 1:10 in ChIP dilution buffer (0.01% SDS, 1.1% Triton X-100, 1.2 mM EDTA, 16.7 mM NaCl, protease inhibitors, 16.7 mM Tris-HCl, pH 8.1). The samples were centrifuged and the recovered chromatin solutions were incubated with 5 μl of indicated antibodies and 24 μl of sonicated salmon sperm (0.1 mg/ml) to remove unspecific background overnight at 4°C with rotation. The antibodies against PPARα (sc-9000), RXRα (sc-553), phosphorylated RNA polymerase II (pPol II, sc-13583) and control IgGs (sc-2027) were obtained from Santa Cruz Biotechnologies (Heidelberg, Germany). The immuno-complexes were collected by incubation with 60 μl of protein A-agarose slurry (Upstate Biotechnology, Lake Placid, NY, USA) for 1 h at 4°C with rotation. The beads were pelleted by centrifugation for 1 minute at 4°C at 100 × g and washed sequentially for 5 minutes by rotation with 1 ml of the following buffers: low salt wash buffer (0.1% SDS, 1% Triton X-100, 2 mM EDTA, 150 mM NaCl, 20 mM Tris-HCl, pH 8.1), high salt wash buffer (0.1% SDS, 1% Triton X-100, 2 mM EDTA, 500 mM NaCl, 20 mM Tris-HCl, pH 8.1) and LiCl wash buffer (0.25 mM LiCl, 1% Nonidet P-40, 1% sodium deoxycholate, 1 mM EDTA, 10 mM Tris-HCl, pH 8.1). Finally, the beads were washed twice with 1 ml of TE buffer (1 mM EDTA, 10 mM Tris-HCl, pH 8.0). The immuno-complexes were then eluted by adding 250 μl elution buffer (1% SDS, 100 mM NaHCO_3_) and incubated for 15 minutes at room temperature with rotation. After centrifugation, the supernatant was collected and the elution was repeated. The supernatants were combined. Subsequently, the cross-linking was reversed and remaining proteins digested by adding proteinase K (final concentration, 80 μg/ml; Fermentas) and incubating overnight at 65°C. Genomic DNA fragments were recovered by phenol-chloroform extraction, followed by a salt-ethanol precipitation and a final re-suspension in sterile H_2_O.

### PCR of chromatin templates

For each of the PPRE-containing genomic regions of the selected PPAR target genes, specific primer pairs were designed (Table 2), optimized and controlled by running PCR reactions with 25 ng of genomic DNA (input) as a template. The Ct values obtained were used to define PCR conditions for output samples. When running immuno-precipitated DNA (output) as a template, the following PCR profile was used: pre-incubation for 5 minutes at 95°C, (Ct_input _+ 10) cycles of 30 s at 95°C, 30 s at 60°C and 45 s at 72°C and one final incubation for 10 minutes at 72°C. PCR product quality was monitored using post-PCR melt curve analysis. The fold inductions were calculated using 2^-(ΔCt)^, where ΔCt is Ct_(specific antibody) _- Ct_(IgG control) _and Ct is the cycle at which the threshold is crossed. Relative association levels were calculated using 2^-(10-Ct(output-input))^.

### Transfection and reporter gene assay

HEK293 and HepG2 cells were seeded into 6-well plates (10^5 ^cells/ml) and grown overnight in phenol red-free DMEM supplemented with 5% charcoal-stripped FBS. Polyethyleneimine transfections were performed by incubating a reporter plasmid and the expression vector for human PPARα, PPARγ or PPARβ/δ (each 1 μg) with 50 μl of 150 mM NaCl for 15 minutes at room temperature. Simultaneously, 15 μg of polyethyleneimine (Sigma-Aldrich) was incubated in 50 μl of 150 mM NaCl. The two solutions were then combined and incubated for an additional 15 minutes at room temperature. After dilution with 900 μl of phenol red-free DMEM, the mixture was added to the cells. Phenol red-free DMEM (500 μl), supplemented with 15% charcoal-stripped FBS and the ligands were added 4 h after transfection. The cells were lysed 16 h later using reporter gene lysis buffer (Roche). The constant light signal *luciferase *reporter gene assay was performed as recommended by the supplier (Perkin-Elmer, Groningen, The Netherlands). Luciferase activities were normalized with respect to protein concentration and induction factors were calculated as the ratio of luciferase activity of ligand-stimulated cells to that of solvent controls.

## Additional data files

The following additional data are available with the online version of this paper. Additional data file 1 is a table of non-binding DR1-type sequences. Additional data file 2 is a table of ten training sets for classifier initializations. Additional data file 3 is a figure comparing the PPRE classifier to matrix methods. Additional data file 4 is a figure of expression profiling of eight validated PPAR target genes in HEK293 and HepG2 cells. Additional data file 5 is a table of the SOM input data set. Additional data file 6 is a listing of further predicted genes from chromosome 19 that have high enrichment of PPREs in human only.

## Supplementary Material

Additional data file 1 Non-binding DR1-type sequences. Click here for file

Additional data file 2 Ten training sets for classifier initializations. Click here for file

Additional data file 3 Comparison of the PPRE classifier to matrix methods. Click here for file

Additional data file 4 Expression profiling of eight validated PPAR target genes in HEK293 and HepG2 cells. Click here for file

Additional data file 5 SOM input data set. Click here for file

Additional data file 6 Further predicted genes from chromosome 19 that have high enrichment of PPREs in human only. Click here for file
