# Supplementary material for: Meta-analysis of primary target genes of peroxisome proliferator-activated receptors
Source: Genome Biol. 2007 Jul 25;8(7):R147. doi: 10.1186/gb-2007-8-7-r147 (PMC2323243; doi:10.1186/gb-2007-8-7-r147)
Supplement: Additional data file 1 — Non-binding DR1-type sequences. [file gb-2007-8-7-r147-S1.pdf]

Additional data file 1: Non-binding DR1-type sequences. DR1-type PPREs that resulted in an average binding gelshift assays for PPAR-RXR heterodimers of less than 1 % in reference to the consensus PPRE **AGGTCAAAGGTCA** are shown (for the stronger PPREs see Table 2). The variations from the consensus PPRE are highlighted in green for PPARα, in dark blue for PPARγ and in light blue for PPARβ/δ. The published PPRE of the *ACOX1* gene [19] is underlined in yellow. SD indicates standard deviation.

PPARα	relative binding	PPARγ	relative binding	PPARδ	relative binding	Mean	SD	Conclusion strong RE
**Category 1/2/0**								
G G T C C A A A G G G C A	0,01	T G G A G A T A G G T C A	0,00	T G G T C A A A G T T C G	0,01	**0,00**	**0,01**	**NF**
A G T T C A A A G T T T A	0,01	G G G T G A A A T G T G C	0,00	G G T C C A A A G G G C A	0,01			
G G G A C A A A G G T T G	0,03	A G G T G A A A A T T C C	0,00	A G T T A A A A G G T T A	0,00			
G G G C C A A G G G T C C	0,00			G G G G A A A A G G T C C	0,00			
A G G G C A A G G G T C T	0,00			A G G G G A A A G G G A A	0,00			
G G G T C A A C G G G T A	0,00			A G G T A A A A T T T C A	0,00			
A G G T A A A A T G T G C	0,00							
**Category 2/2/0**								
A T C T T A A G G G T C A	0,00	A T C T T A A G G G T C A	0,00	T G G A G A T A G G T C A	0,00	**0,00**	**0,01**	**NF**
A G T G G A A A G G T A A	0,00	A G G A C A A G G G G C T	0,00	A T C T T A A G G G T C A	0,00			
A G G A C A A G G G G C T	0,00	A G G G C A T C A G T C A	0,01	A G G T A A A A T G T G C	0,00			
G G G G C A A A G G G T G	0,02	A G G T A A A A T G T G C	0,00	A G G T G A A A A T T C C	0,00			
A G G T G A A A A T T C C	0,00			G G G T G A A A T G T G C	0,00			
G G G T G A A A T G T G C	0,00							
A G G T T A A G T G T G A	0,00							
G G G T C A A G T G G T A	0,01							
**Category 1/3/0**								
G G G C T A A A T G T G A	0,00			G G G A G A A A G G G A A	0,00	**0,00**	**0,00**	**NF**
A G G C C A A G G G T T C	0,00			A G G A C A A G G G G C T	0,00			
A G G G C A T C A G T C A	0,00			G G G T G A A A G G G A C	0,00			
				A G G T C A A A G T C A C	0,00			
**Category 4/1/0**								
A G G T G A A A G T G T G	0,00	A G T T T A T A G T T G A	0,00	A G T T T A T A G T T G A	0,00	**0,00**	**0,00**	**NF**
		A G G C C A T G G G T T C	0,00	A G G T T A T G C T T C A	0,00			
		A G G T T A T G C T T C A	0,00	A G G C C A T G G G T T C	0,00			
		A G G T G A T G G G C A C	0,00					
**Category 2/0/1**								
A G G T G G A A G G T A A	0,00	A G G G G A A G G G A C A	0,00	T G G T T A A A G G T T A	0,01	**0,00**	**0,01**	**NF**
A G G T G A G A G G T G A	0,01	A G G G C A A A G T A C A	0,01	A G G G C A A A G T A C A	0,00			
				G G G T G A G A G G T G A	0,00			
**Category 1/1/1**								
A G G G C A A A G T A C A	0,01			A G C T G A A A G G T A A	0,00	**0,00**	**0,01**	**NF**
				A G G T G G A A G G T A A	0,00			
				G G G T C A A C G G G T A	0,00			
**Category 0/3/0**								
A G G T C A G C T G T C A	0,00			G T G T A A A A G G T C G	0,00	**0,00**	**0,00**	**NF**
				A G G A A A A A G G C C A	0,00			
**Category 3/0/1**								
G G G T C A T A G G A G G	0,00	A G G G C G A A G T C C A	0,00	A G G G G A A G G G A C A	0,00	**0,00**	**0,00**	**NF**
		G G G T C A T A G G A G G	0,00					
**Category 2/2/1**								
		G T G T A G A A A G T C G	0,00	A G C A C T T G G G T C A	0,00	**0,00**	**0,00**	**NF**
		A G C A C T T G G G T C A	0,00					
**Category 2/3/0**								
C G C C C A G A G T T C A	0,00	G A G T G A T G T G T C T	0,00	A G G G G A A A G G C A C	0,00	**0,00**	**0,00**	**NF**
A G G C C A T G G G T T C	0,00			A G G T G A A A G T G T G	0,00			
A G G G G A A A G G C A C	0,00							
**Category 2/1/1**								
A G G G G A A G G G A C A	0,00			T G G T G A A A G T T A A	0,00	**0,00**	**0,00**	**NF**
A G G G C G A A G T C C A	0,00			T G G T G A A A G G T G G	0,00			
				A G C T G A A A G T T A A	0,00			
				A G G G C A T C A G T C A	0,00			
				G G G T C A T A G G A G G	0,00			
				A G G G C G A A G T C C A	0,00			
**Category 3/2/0**								
A G T T T A T A G T T G A	0,00					**0,00**	**0,00**	**NF**
A G G G G A A A G G C A C	0,00							
A G G T T A T G C T T C A	0,00							
**Category 3/3/0**								
G A G T G A T G T G T C T	0,00			G A G T G A T G T G T C T	0,00	**0,00**	**0,00**	**NF**
				A G G T G A T G G G C A C	0,00			
**Category 0/2/1**								
		A G G T C A G C T G T C A	0,00	A G G T C A G C T G T C A	0,00	**0,00**	**0,00**	**NF**
