# Supplementary material for: Meta-analysis of primary target genes of peroxisome proliferator-activated receptors
Source: Genome Biol. 2007 Jul 25;8(7):R147. doi: 10.1186/gb-2007-8-7-r147 (PMC2323243; doi:10.1186/gb-2007-8-7-r147)
Supplement: Additional data file 2 — Ten training sets for classifier initializations. [file gb-2007-8-7-r147-S2.pdf]

Additional data file 2: Ten initializations of a PPRE classifier by random sampling. Ten rounds of random sampling were performed to create training sets (10 % of data) and validation sets for each classifier instance. Category averages calculated based on the training sets are shown.

**Category**	**N1**	**N2**	**N3**	**N4**	**N5**	**N6**	**N7**	**N8**	**N9**	**N10**	**average**
**1/0/0**	0.62	0.71	0.62	0.68	0.67	0.65	0.69	0.71	0.66	0.74	**0.68**
**0/1/0**	0.45	0.4	0.46	0.46	0.39	0.47	0.44	0.50	0.40	0.45	**0.44**
**2/0/0**	0.27	0.26	0.6	0.26	0.25	0.5	0.21	0.25	0.50	0.50	**0.36**
**3/0/0**	0.15	0.24	0.09	0.35	0.25	0.09	0.18	0.18	0.6	0.09	**0.22**
**1/1/0**	0.14	0.26	0.28	0.13	0.17	0.26	0.19	0.19	0.19	0.17	**0.20**
**0/0/1**	0.22	0.28	0.15	0.09	0.19	0.18	0.29	0.26	0.19	0.15	**0.20**
**0/2/0**	0.09	0.00	0.13	0.00	0.09	0.06	0.09	0.09	0.00	0.18	**0.07**
**2/1/0**	0.04	0.05	0.01	0.06	0.03	0.04	0.06	0.07	0.07	0.02	**0.05**
**1/0/1**	0.02	0.02	0.06	0.02	0.06	0.01	0.06	0.01	0.02	0.02	**0.03**
**3/1/0**	0.00	0.01	0.00	0.01	0.00	0.01	0.05	0.03	0.03	0.03	**0.02**
**4/0/0**	0.02	0.04	0.02	0.02	0.00	0.04	0.00	0.04	0.02	0.04	**0.02**
**1/2/0**	0.01	0.00	0.02	0.01	0.00	0.01	0.00	0.01	0.01	0.02	**0.01**
**2/2/0**	0.00	0.01	0.00	0.00	0.01	0.00	0.00	0.00	0.00	0.00	**0.00**
**1/3/0**	0.00	0.00	0.00	0.00	0.00	0.00	0.00	0.00	0.00	0.00	**0.00**
**4/1/0**	0.00	0.00	0.00	0.00	0.00	0.00	0.00	0.00	0.00	0.00	**0.00**
**2/0/1**	0.01	0.01	0.01	0.00	0.01	0.00	0.00	0.01	0.01	0.01	**0.01**
**1/1/1**	0.00	0.00	0.00	0.01	0.00	0.00	0.00	0.00	0.01	0.01	**0.00**
**0/3/0**	0.00	0.00	0.00	0.00	0.00	0.00	0.00	0.00	0.00	0.00	**0.00**
**3/0/1**	0.00	0.00	0.00	0.00	0.00	0.00	0.00	0.00	0.00	0.00	**0.00**
**2/2/1**	0.00	0.00	0.00	0.00	0.00	0.00	0.00	0.00	0.00	0.00	**0.00**
**2/3/1**	0.00	0.00	0.00	0.00	0.00	0.00	0.00	0.00	0.00	0.00	**0.00**
**2/1/1**	0.00	0.00	0.00	0.00	0.00	0.00	0.00	0.00	0.00	0.00	**0.00**
**3/2/0**	0.00	0.00	0.00	0.00	0.00	0.00	0.00	0.00	0.00	0.00	**0.00**
**3/3/0**	0.00	0.00	0.00	0.00	0.00	0.00	0.00	0.00	0.00	0.00	**0.00**
**0/2/1**	0.00	0.00	0.00	0.00	0.00	0.00	0.00	0.00	0.00	0.00	**0.00**
