# Supplementary material for: Meta-analysis of primary target genes of peroxisome proliferator-activated receptors
Source: Genome Biol. 2007 Jul 25;8(7):R147. doi: 10.1186/gb-2007-8-7-r147 (PMC2323243; doi:10.1186/gb-2007-8-7-r147)
Supplement: Additional data file 3 — Comparison of the PPRE classifier to matrix methods. [file gb-2007-8-7-r147-S3.pdf]

Additional data file 3: Comparison of PPRE classifier to matrix methods. The performance of different PPRE search methods was compared on the basis of the PPARγ data in Tables 2 and Additional data file 1. A position-specific weight matrix was constructed from 20 medium and strong PPREs that contain multiple variations, a position-specific affinity matrix was constructed using the single nucleotide data and a PPRE classifier was created based on Table 1. The three methods were used to score the data in Table 2 and Additional data file 1 separately for PPREs containing single (A) or multiple variations (B) and PPREs having an average binding strength below 1 (C). According to the base independence assumption linear curves were fitted to the data points to compare the performance of the methods. The lines fitted to single nucleotide data were also superimposed on the multiple variation data (black line).

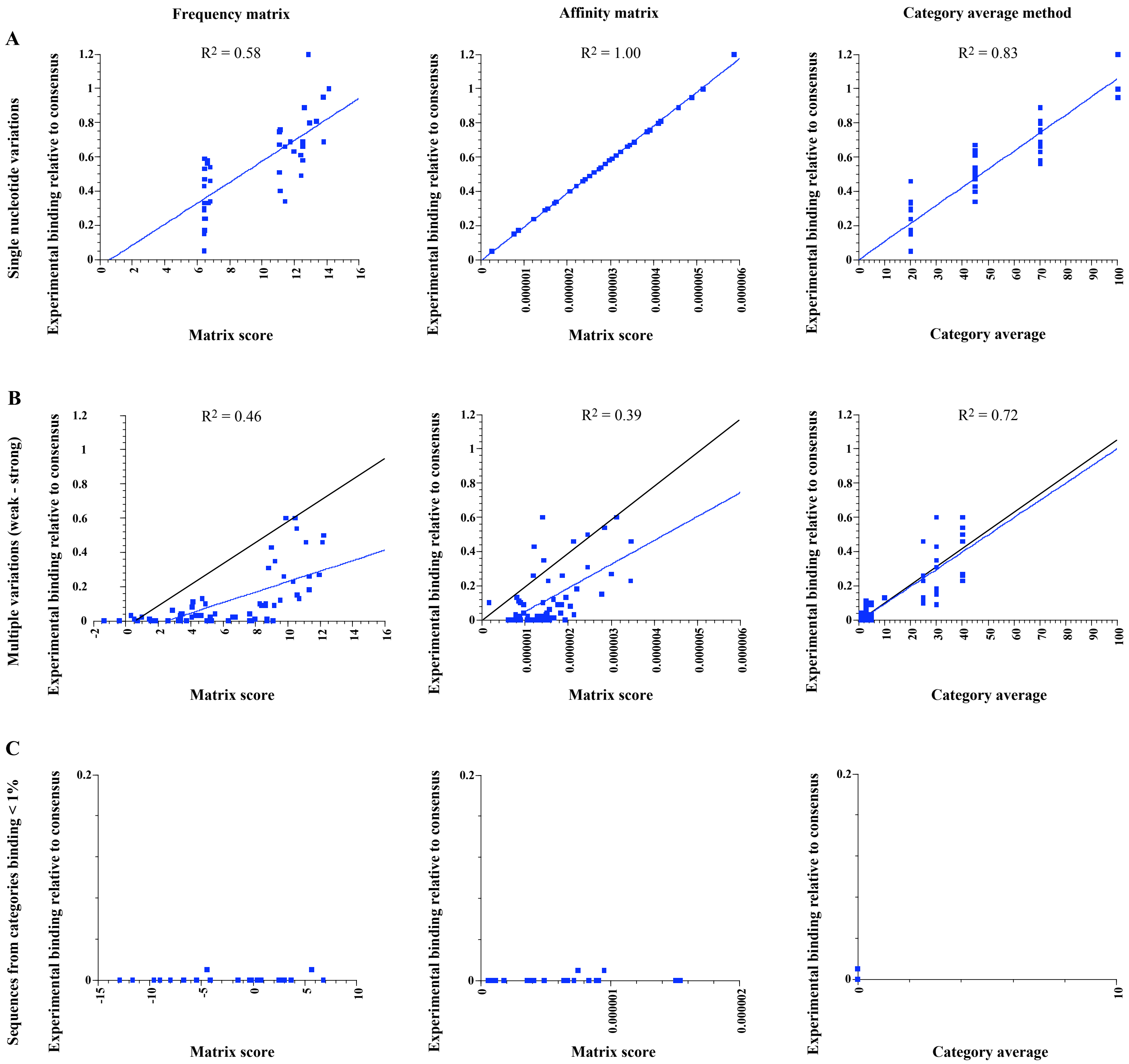
Frequency matrix
Affinity matrix
Category average method
A
Single nucleotide variations
B
Multiple variations (weak - strong)
C
Sequences from categories binding < 1%
Experimental binding relative to consensus
Matrix score
Category average
R2 = 0.58
R2 = 1.00
R2 = 0.83
R2 = 0.46
R2 = 0.39
R2 = 0.72
