# Supplementary material for: Meta-analysis of primary target genes of peroxisome proliferator-activated receptors
Source: Genome Biol. 2007 Jul 25;8(7):R147. doi: 10.1186/gb-2007-8-7-r147 (PMC2323243; doi:10.1186/gb-2007-8-7-r147)
Supplement: Additional data file 4 — Expression profiling of eight validated PPAR target genes in HEK293 and HepG2 cells. [file gb-2007-8-7-r147-S4.pdf]

Additional data file 4. Expression profiling of selected PPAR target genes. Real-time quantitative PCR was used to determine the inducibility of the mRNA expression of the indicated eight PPAR target genes, relative to the control gene *RPLP0*, in HEK293 (A-C) and HepG2 (D-F) cells. Please note that the *APOC3* gene is not expressed in HEK293 cells. The cells were stimulated for 2, 4 and 6 h with 100 nM of the PPARα ligand GW7647 (A and D), 100 nM of the PPARγ ligand rosiglitazone (B and E) or 100 nM of the PPARβ/δ ligand GW501516 (C and F). Columns represent the means of at least three independent treatments and the bars represent standard deviations. Two-tailed Student's t-tests were performed to determine the significance of the mRNA induction by PPAR agonists in reference to solvent controls (* $p < 0.05$, ** $p < 0.01$, *** $p < 0.001$).

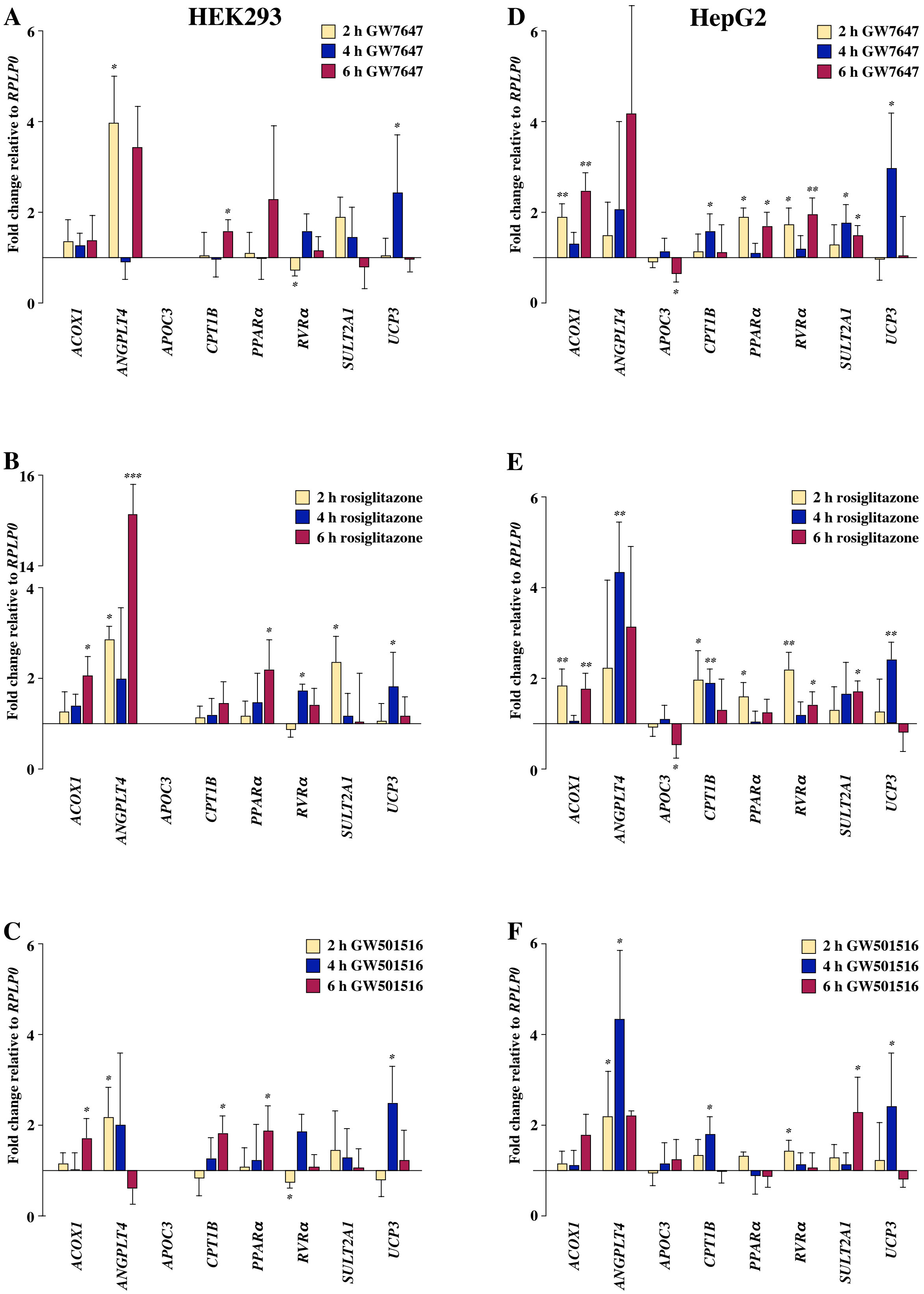

A
HEK293
D
HepG2
B
E
C
F
Fold change relative to RPLP0
2 h GW7647
4 h GW7647
6 h GW7647
2 h rosiglitazone
4 h rosiglitazone
6 h rosiglitazone
2 h GW501516
4 h GW501516
6 h GW501516
ACOX1
ANGPLT4
APOC3
CPT1B
PPARα
RVRα
SULT2A1
UCP3
