# Supplementary material for: Meta-analysis of primary target genes of peroxisome proliferator-activated receptors
Source: Genome Biol. 2007 Jul 25;8(7):R147. doi: 10.1186/gb-2007-8-7-r147 (PMC2323243; doi:10.1186/gb-2007-8-7-r147)
Supplement: Additional data file 5 — SOM input data set. [file gb-2007-8-7-r147-S5.pdf]

Additional data file 5: Input dataset for SOM analysis. The variable BS represents the sum of predicted binding strength of n putative medium or strong PPREs (Σbs_n, $bs_i = \max\{bs_{PPAR\alpha}, bs_{PPAR\gamma}, bs_{PPAR\beta/\delta}\}$) found within 20 kB the analyzed region of each gene in human (h) and mouse (m), respectively. The remaining values indicate the number of conserved strong/medium (CS) or weak (CW) PPREs in human and mouse.

Cluster	Gene	BS_h	BS_m	CS_h	CW_h	CS_m	CW_m
**IA**	*ANGPLT4*	0.600	0.386	1	1	1	1
	CPT1A	1.000	0.140	1	1	1	1
	PEPCK1	0.600	0.561	1	0	1	1
	LPL	0.350	0.421	1	1	1	0
**IB**	*GK*	0.450	0.175	1	1	0	1
	UCP3	0.400	0.491	1	1	0	1
**IC**	*LRP1*	0.600	0.632	0	1	1	1
**ID**	*caveolin 1*	0.000	0.211	0	1	1	1
	IGFBP1	0.150	0.351	0	1	1	1
**IIA**	*APOC3*	0.500	0.596	1	0	1	0
	CPT1B	0.550	0.509	1	0	1	0
	CPT2	0.500	0.211	1	0	1	0
	CYP1A1$_E$	0.650	0.246	1	0	0	0
	CYP1A1$_N$	0.650	0.421	1	0	1	0
	HMGCS2	0.575	0.281	1	0	1	0
	SRB1	0.500	0.316	1	0	1	0
**IIB**	*ADRP*	0.200	0.316	1	0	1	0
	APOA1	0.300	0.632	1	0	1	0
	G0S2	0.250	0.281	1	0	1	0
	LXRα	0.300	0.421	1	0	1	0
	SSAT	0.200	0.386	1	0	1	0
**IIIA**	*COX2*	0.211	0.105	1	0	0	1
	SEMA6B	0.070	0.596	1	0	0	1
**IIIB**	*APOA2*	0.450	0.421	0	1	0	1
	APOA5	0.700	0.105	0	1	0	1
	FADS2$_E$	0.200	0.211	0	0	0	0
	FADS2$_N$	0.000	0.211	0	1	0	1
	PXR	0.250	0.211	0	1	0	1
	RVRα	0.200	0.298	0	1	0	1
	SLC10A2	0.600	0.105	0	1	0	1
**IVA**	*ACOX1*	0.550	1.000	0	0	0	0

**IVB**	*Resistin*	0.300	0.246	0	0	0	0
	SULT2A1	0.350	0.404	0	0	0	0
**IVC**	*APOE*	0.500	0.105	0	0	0	0
	PPARα	0.350	0.175	0	0	0	0
	CYP27A1	0.350	0.105	0	0	0	0
**IVD**	*GSTA2*	0.100	0.281	0	0	0	0
	transferrin	0.100	0.211	0	0	0	0
	UGT1A9	0.100	0.175	0	0	0	0
**IVE**	*CDKN2A*	0.000	0.140	0	0	0	0
