# Supplementary material for: Meta-analysis of primary target genes of peroxisome proliferator-activated receptors
Source: Genome Biol. 2007 Jul 25;8(7):R147. doi: 10.1186/gb-2007-8-7-r147 (PMC2323243; doi:10.1186/gb-2007-8-7-r147)
Supplement: Additional data file 6 — Further predicted genes from chromosome 19 that have high enrichment of PPREs in human only. [file gb-2007-8-7-r147-S6.pdf]

Additional data file 6: Further predicted PPAR target genes in human chr 19. All 1445 genes of human chr 19 were screened *in silico* for strong and medium PPREs. All putative PPRE sequences are available on request. The 75 genes that carry three or more strong or medium PPREs (where at least one is strong) are listed. Further 50 genes were already identified in the first chr 19 screening approach (highlighted bold in Table 5).

**Ensembl ID**	**Name**
ENSG00000005206	*Signal peptide peptidase-like 2B*
ENSG00000006659	*Galectin-14*
ENSG00000011451	*WIZ protein*
ENSG00000064607	*Putative splicing factor, arginine/serine rich 14*
ENSG00000064666	*Calponin-2*
ENSG00000068903	*sirtuin-2*
ENSG00000070388	*Fibroblast growth factor 22 precursor*
ENSG00000079432	*Protein capicua homolog*
ENSG00000080573	*Collagen* α*-3(V) chain precursor*
ENSG00000083817	*Zinc finger protein 416*
ENSG00000090920	*Fc fragment of IgG binding protein*
ENSG00000099804	*Ubiquitin-conjugating enzyme E2-32 kDa complementing*
ENSG00000099821	*DNA-directed RNA polymerase, mitochondrial precursor*
ENSG00000104774	*Lysosomal* α*-mannosidase precursor*
ENSG00000104825	*NF-kappa-B inhibitor* β
ENSG00000104833	*Tubulin* β*-4 chain*
ENSG00000104892	*Kinesin light chain 3*
ENSG00000105063	*SAPS domain family member 1*
ENSG00000105202	*rRNA 2'-O-methyltransferase fibrillarin*
ENSG00000105270	*Cytoplasmic linker protein 170-related 59 kDa protein*
ENSG00000105325	*Fizzy-related protein homolog*
ENSG00000105357	*Myosin-14*
ENSG00000105366	*Sialic acid-binding Ig-like lectin 8 precursor*
ENSG00000105373	*Glioma tumor suppressor candidate region gene 2 protein*
ENSG00000105507	*Calcium-binding protein 5*
ENSG00000105605	*Voltage-dependent calcium channel gamma-7 subunit*
ENSG00000105676	*Armadillo repeat-containing protein 6*
ENSG00000131037	*Epidermal growth factor receptor kinase substrate 8-like protein 1*
ENSG00000195711	*Sodium channel beta-1 subunit precursor*
ENSG00000124444	*Zinc finger protein 576*
ENSG00000125650	*Persephin precursor*
ENSG00000125652	*Alkylated repair protein alkB homolog 7 precursor*
ENSG00000125730	*Complement C3 precursor*
ENSG00000126464	*Proline-rich protein 12*

ENSG00000127663 *JmjC domain-containing histone demethylation protein 3B*
ENSG00000130283 ***LASS1***
ENSG00000130332 *U6 snRNA-associated Sm-like protein LSm7*
ENSG00000130433 *Voltage-dependent calcium channel gamma-6 subunit*
ENSG00000130477 *Unc-13 homolog A*
ENSG00000130513 *Growth/differentiation factor 15 precursor*
ENSG00000130725 *NEDD8-conjugating enzyme Ubc12*
ENSG00000130876 *Asc-type amino acid transporter 1*
ENSG00000132026 *Retbindin*
ENSG00000142235 *LMTK3 protein (Fragment)*
ENSG00000142512 *Sialic acid-binding Ig-like lectin 10 precursor*
ENSG00000160296 *Sialic acid-binding Ig-like lectin 12 precursor*
ENSG00000161265 *U2 small nuclear RNA auxiliary factor 1-like 4 isoform 1*
ENSG00000167378 *Immunity-related GTPase family, Q1*
ENSG00000167748 *Kallikrein-1 precursor*
ENSG00000169035 *Kallikrein-7 precursor*
ENSG00000174677 *Vomeronasal type-1 receptor 4*
ENSG00000176490 *GTP-binding protein Di-Ras1*
ENSG00000177202 *Sperm acrosomal membrane protein 14*
ENSG00000177464 *Probable G-protein coupled receptor 4*
ENSG00000181029 *Trafficking protein particle complex subunit 5*
ENSG00000182087 *Membralin*
ENSG00000183019 *Mast cell-expressed membrane protein 1*
ENSG00000184771 *CCAAT/enhancer binding protein*
ENSG00000186526 ***CYP4F8***
ENSG00000187534 *Proline-rich protein 13*
ENSG00000187244 *Lutheran blood group glycoprotein precursor*
ENSG00000187664 *Hyaluronan and proteoglycan link protein 4 precursor*
ENSG00000188223 *Antolefinin*
ENSG00000188293 *Insulin growth factor-like family member 1 precursor*
ENSG00000188321 *Zinc finger protein 559*
ENSG00000189114 *Biogenesis of lysosome-related organelles complex-1 subunit 3*
ENSG00000189377 *DMC*
ENSG00000196131 *Vomeronasal type-1 receptor 2*
ENSG00000196442 *Zinc finger protein 432*
ENSG00000196826 *Zinc finger protein 709*
ENSG00000196967 *Zinc finger protein 585A*
ENSG00000196988 *HERV-H_19p13.11 provirus ancestral Env polyprotein precursor*
ENSG00000197128 *Zinc finger protein 772*
ENSG00000197483 *Zinc finger protein 628*
ENSG00000204869 *Insulin growth factor-like family member 4 precursor*
